# Phosphatidylinositol Kinases and Phosphatases in *Entamoeba histolytica*

**DOI:** 10.3389/fcimb.2019.00150

**Published:** 2019-06-06

**Authors:** Kumiko Nakada-Tsukui, Natsuki Watanabe, Tomohiko Maehama, Tomoyoshi Nozaki

**Affiliations:** ^1^Department of Parasitology, National Institute of Infectious Diseases, Tokyo, Japan; ^2^Graduate School of Life and Environmental Sciences, University of Tsukuba, Tsukuba, Japan; ^3^Division of Molecular and Cellular Biology, Graduate School of Medicine, Kobe University, Kobe, Japan; ^4^Department of Biomedical Chemistry, Graduate School of Medicine, The University of Tokyo, Tokyo, Japan

**Keywords:** *Entamoeba histolytica*, phosphoinositide, kinase, phosphatase, signaling

## Abstract

Phosphatidylinositol (PtdIns) metabolism is indispensable in eukaryotes. Phosphoinositides (PIs) are phosphorylated derivatives of PtdIns and consist of seven species generated by reversible phosphorylation of the inositol moieties at the positions 3, 4, and 5. Each of the seven PIs has a unique subcellular and membrane domain distribution. In the enteric protozoan parasite *Entamoeba histolytica*, it has been previously shown that the PIs phosphatidylinositol 3-phosphate (PtdIns3P), PtdIns(4,5)P_2_, and PtdIns(3,4,5)P_3_ are localized to phagosomes/phagocytic cups, plasma membrane, and phagocytic cups, respectively. The localization of these PIs in *E. histolytica* is similar to that in mammalian cells, suggesting that PIs have orthologous functions in *E. histolytica*. In contrast, the conservation of the enzymes that metabolize PIs in this organism has not been well-documented. In this review, we summarized the full repertoire of the PI kinases and PI phosphatases found in *E. histolytica* via a genome-wide survey of the current genomic information. *E. histolytica* appears to have 10 PI kinases and 23 PI phosphatases. It has a panel of evolutionarily conserved enzymes that generate all the seven PI species. However, class II PI 3-kinases, type II PI 4-kinases, type III PI 5-phosphatases, and PI 4P-specific phosphatases are not present. Additionally, regulatory subunits of class I PI 3-kinases and type III PI 4-kinases have not been identified. Instead, homologs of class I PI 3-kinases and PTEN, a PI 3-phosphatase, exist as multiple isoforms, which likely reflects that elaborate signaling cascades mediated by PtdIns(3,4,5)P_3_ are present in this organism. There are several enzymes that have the nuclear localization signal: one phosphatidylinositol phosphate (PIP) kinase, two PI 3-phosphatases, and one PI 5-phosphatase; this suggests that PI metabolism also has conserved roles related to nuclear functions in *E. histolytica*, as it does in model organisms.

## 1. Introduction

Phosphoinositides (PIs) are phosphorylated-phosphatidylinositol (PtdIns) derivatives and play pivotal roles in a variety of biological processes such as receptor-mediated signaling, vesicular traffic, cytoskeleton rearrangement, and regulation of channels and transporters (Sasaki et al., 2009; Balla, 2013). Spatiotemporal regulation of PI-mediated biological processes is achieved by interconversion of the phosphorylation states of PIs by specific kinases and phosphatases, followed by recruitment of PI-specific effectors. Phospholipids are ubiquitous in all three domains of life. Nevertheless, the complexity of PIs and enzymes that interconvert them appears to have increased in eukaryotes (Michell, 2008, 2011). It has been suggested that the PI metabolism developed in the last common eukaryotic ancestor (Michell, 2008) and diverged during eukaryotic evolution.

Human amebiasis is a common infection caused by the protozoan parasite *Entamoeba histolytica* in both developing and developed countries (Taniuchi et al., 2013; Lo et al., 2014; Ishikane et al., 2016), causing as far as 73,800 deaths annually (Lozano et al., 2012). The transmission usually occurs upon ingestion of water or food contaminated with *E. histolytica* cysts. The ingested cysts pass through the stomach and differentiate into trophozoites that colonize the colon. It is estimated that only 10–20% of individuals who are infected with *E. histolytica* develop symptoms (Gathiram and Jackson, 1985; Marie and Petri, 2014). The most common clinical manifestations in symptomatic cases are colitis and dysentery, and 5–10% of these are accompanied by invasive extraintestinal amebiasis, which is mostly amoebic liver abscess (Walsh, 1986).

*Entamoeba histolytica* belongs to the eukaryotic supergroup Amoebozoa, which is only distantly related to the eukaryotic model organisms in the Opisthokonta clade, including *Saccharomyces cerevisiae, Caenorhabditis elegans, Drosophila melanogaster*, and *Homo sapiens*. Various unique features of *E. histolytica* have been described due to its anaerobic/microaerophilic and parasitic life style, including metabolism of sulfur-containing amino acids, anaerobic energy generation, anti-oxidative stress mechanisms, and compartmentalization of sulfate activation to mitosomes, a unique mitochondria-related organelle (Ali and Nozaki, 2007; Müller et al., 2012; Makiuchi and Nozaki, 2014; Jeelani and Nozaki, 2016; Mi-Ichi et al., 2017; Pineda and Perdomo, 2017). Furthermore, the mechanisms regulating membrane trafficking in *E. histolytica* appear to be at least as complex as those found in higher eukaryotes. While most of the machineries underlying membrane-trafficking such as clathrin coats, coatomers, SNAREs, ESCRTs, and the retromer complex are conserved in *E. histolytica* (Nakada-Tsukui et al., 2005; Clark et al., 2007; Leung et al., 2008), unique evolutionary features in membrane trafficking are also apparent. For example, *E. histolytica* has numerous extremely diversified Rab small GTPases (104 genes) despite its unicellularity throughout its life cycle (Saito-Nakano et al., 2005; Nakada-Tsukui et al., 2010). In addition, a family of unique receptors that transport lysosomal hydrolase emerged in *Entamoeba* and related lineages during evolution (Furukawa et al., 2012, 2013; Nakada-Tsukui et al., 2012; Marumo et al., 2014). Although membrane trafficking in *E. histolytica* has been well-studied in the last few decades, *E. histolytica* PIs and PI metabolism are still relatively elusive despite the fact that they likely play critical roles in the physiology, especially in membrane trafficking, and pathogenicity of this organism (Raha et al., 1994, 1995; Giri et al., 1996; Makioka et al., 2001; Powell et al., 2006; Blazquez et al., 2008; Nakada-Tsukui et al., 2009; Byekova et al., 2010; Goldston et al., 2012; Koushik et al., 2013, 2014; López-Contreras et al., 2013; Lee et al., 2014; Bharadwaj et al., 2017). A previous genome-wide survey suggested that PI effectors found in other eukaryotes are not well-conserved in *E. histolytica* (Nakada-Tsukui et al., 2009). In this particular study, in order to better understand the level of conservation, elimination or diversification of the enzymes involved in the metabolism of *E. histolytica* PIs, we performed an extensive search for the potential kinases and phosphatases specific for the PIs found in the genome of this pathogen. Additionally, we summarized the known structural features and functions of similar enzymes in other organisms. To find and weigh the significance of possible homologs, we primarily used the E-values in the BLAST search. This was because *E. histolytica* homologs often differ in domain configurations and protein lengths to homologs in model organisms and the E-values better reflect both local and entire protein similarity. Such a comprehensive understanding of PI kinases and phosphatases will help us construct new hypotheses in future research.

## 2. General Overview on Intracellular Localization and Roles of PIs

### 2.1. Definition, Structure, Synthesis, Transport, and Localization of PIs

#### 2.1.1. Definition, Structure, Synthesis, and Transport of PIs

**Figure 1:**
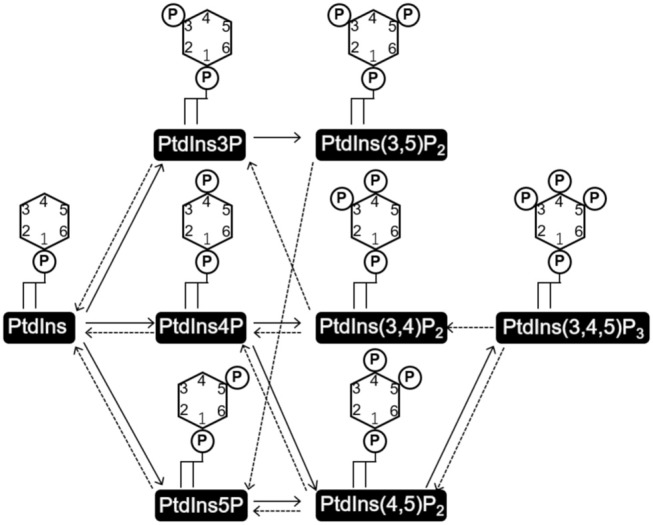
Structures of phosphatidylinositol (PtdIns) and phosphoinositides (PI), and the routes of their interconversion. PtdIns consists of a glycerol backbone with two covalently attached fatty acids at the *sn-1* and *sn-2* positions, and a D-*myo*-inositol head group linked via the phosphate at the *sn-3* position. Three hydroxyl groups of the D-*myo*-inositol head group (D3-5) are independently phosphorylated or dephosphorylated to form the seven kinds of phosphorylated PtdIns, PIs. Solid and broken arrows indicate kinase and phosphate reactions, respectively.

PtdIns consists of a glycerol backbone with two covalently bound fatty acids at the stereospecifically numbered (*sn*)-1 and 2 positions, and a D-*myo*-inositol head group linked via the *sn*-3 phosphate of glycerol. Three hydroxyl groups of the D-*myo*-inositol head group (D3–5) are independently phosphorylated or dephosphorylated to form seven kinds of phosphorylated PtdIns (PIs) (Figure 1). PtdIns is synthesized in the endoplasmic reticulum (ER) from cytidine diphosphate diacylglycerol (CDP-DAG) and *myo*-inositol by PtdIns synthase (PIS) and transported to other cellular compartments either by vesicular transport or by PI transfer proteins (PITPs) (Di Paolo and De Camilli, 2006; Lev, 2010; Das and Nozaki, 2018). PtdInss are further metabolized to a variety of PIs on the membranes of these organelles (Figure 1).

#### 2.1.2. Localization of PIs

**Figure 2:**
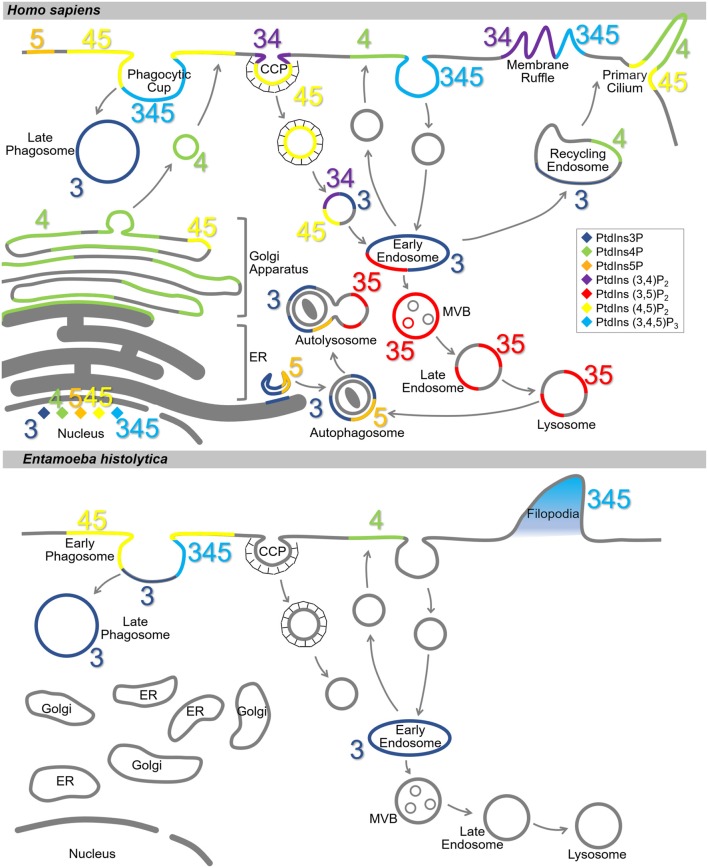
Subcellular localization of PIs in *Homo sapiens* and *Entamoeba histolytica*. Schematic representation of subcellular localization of PIs in *H. sapiens* **(Upper)** and *E. histolytica* **(Lower)**. The seven PI species are depicted with different colors as indicated in the islet. Numbers 3, 4, and 5 depict the positions of phosphates in the PIs. Note that localization of the PIs in *E. histolytica* is largely elusive. CCP, clathrin-coated pit; MVB, multi vesicular body; ER, endoplasmic reticulum.

PtdIns and PIs are concentrated at the cytosolic surface of the plasma membrane. Each PI type is enriched in a specific compartment(s) or sub-compartment(s) (Balla, 2013; Schink et al., 2016) (Figure 2). This disequilibrium in the type and distribution of PIs serves as a molecular tag to recruit specific effectors (Hammond and Balla, 2015; Várnai et al., 2017). In the model organisms, the distribution of PtdIns and PIs has been well-characterized. PtdIns4P and PtdIns(4,5)P_2_ are enriched on the plasma membrane, where PtdIns(3,4)P_2_ and PtdIns(3,4,5)P_3_ are transiently generated *in situ* in response to extracellular stimuli or intracellular signaling (Di Paolo and De Camilli, 2006). PtdIns4P is enriched in the Golgi apparatus, where it regulates both intra-Golgi trafficking and the subsequent transport to the plasma membrane or the endosomal system (De Matteis et al., 2013). PtdIns3P is enriched in early endosomes and is known to trigger the recruitment of a number of effector proteins important for early endosomal identity and function (Di Paolo and De Camilli, 2006; Marat and Haucke, 2016; Schink et al., 2016). PtdIns(3,5)P_2_, converted from PtdIns3P, accumulates in the multivesicular bodies (MVBs) and late endosomes/lysosomes as early endosomes mature (Marat and Haucke, 2016). PtdIns5P is present in the nucleus, plasma membrane, and endomembranes including autophagosomes (Hammond and Balla, 2015; Vicinanza et al., 2015; Várnai et al., 2017), and functions in cytoskeleton regulation, and stress signaling pathways (Viaud et al., 2014). Except for PtdIns(3,4)P_2_ and PtdIns(3,5)P_2_, nuclear localization of all the PIs has been reported (Ye and Ahn, 2008). Although PI metabolism in the nucleus is not fully understood, the involvement of nuclear PIs in transcription and chromatin remodeling in mammals, fly, yeast, and plant has been reported (Cheng and Shearn, 2004; Blind et al., 2012; Dieck et al., 2012; Shah et al., 2013; Poli et al., 2016).

### 2.2. Physiological Roles of PIs

#### 2.2.1. Signaling via Phospholipase C-PtdIns(4,5)P_2_ Breakdown

PIs are involved in signaling via two major pathways: as precursors of second messengers, and as regulators of various PI-specific effectors. The role of phospholipase C (PLC), which breaks down PtdIns(4,5)P_2_ to inositol 1,4,5-trisphosphate[Ins(1,4,5)P_3_] and DAG, in the receptor-mediated growth signal pathway was first demonstrated in the early '80s (Michell et al., 1981; Berridge, 1983; Nishizuka, 1984; Michell, 1995). The role of PI turnover and PI-mediated signaling in cell proliferation is well-established (Berridge, 1984, 1987). PI turnover has also been implicated in the upstream signaling of Ca^2+^ fluxes (Fain and Berridge, 1979). Given that the primary target of PLC is PtdIns(4,5)P_2_ but not PtdIns (Berridge, 1983; Berridge et al., 1983; Creba et al., 1983), and Ins(1,4,5)P_3_ is involved in Ca^2+^ release from the ER, PLC and PtdIns(4,5)P_2_ indirectly affect the regulation of non-mitochondrial Ca^2+^ storage (Streb et al., 1983, 1984; Volpe et al., 1985). DAG activates the phospholipid-dependent kinase family, protein kinase C (PKC), and subsequently, the downstream signaling cascades (Nishizuka, 1984, 1995).

Ins(1,4,5)P_3_-mediated calcium signaling is conserved in a wide range of eukaryotes (Plattner and Verkhratsky, 2013). Interestingly, Ins(1,4,5)P_3_ can be generated by an alternative pathway independent of PLC, and many protists do not have orthologous genes for the canonical Ins(1,4,5)P_3_ receptor, which regulates Ca^2+^ release from the ER (Kortholt et al., 2007; Plattner and Verkhratsky, 2013; Artemenko et al., 2014; Garcia et al., 2017). However, Ca^2+^ release by Ins(1,4,5)P_3_ has been observed even in the organisms without an Ins(1,4,5)P_3_ receptor. Besides, an orthologous gene has been identified in *Trypanosoma cruzi*, which is responsible for Chagas disease, suggesting some extent of conservation of the signaling pathway among eukaryotes (Hashimoto et al., 2013; Plattner and Verkhratsky, 2013).

#### 2.2.2. Vesicular Traffic

PIs are involved in a variety of processes that involve vesicular trafficking, including secretion, recycling, endocytosis/phagocytosis, and autophagy (Frere et al., 2012; Balla, 2013; Swanson, 2014; Klinkert and Echard, 2016; Makowski et al., 2017; Wallroth and Haucke, 2018). A majority of secretory proteins are first transported into the ER lumen through the translocon on the ER membrane, then to the Golgi, where they are packaged into transport vesicles to be dispatched to endosomes or the plasma membrane. PtdIns4P, which is enriched in the Golgi, cooperatively works with PtdIns4P effectors such as GGA (Golgi-localized, gamma adaptin ear-containing, ARF-binding; a clathrin adaptor protein), Arf1, Ypt32p/Rab11 (small GTPase), and Sec2 (RabGEF) to form and target transport vesicles to the plasma membrane (De Matteis et al., 2013; Makowski et al., 2017). At the plasma membrane, PtdIns(4,5)P_2_ cooperates with its effectors and promotes fusion of secretory vesicles with the plasma membrane (Li and Chin, 2003; Balla, 2013; Martin, 2015). PtdIns(4,5)P_2_ at the plasma membrane is involved in the initiation of internalization processes such as endocytosis, micropinocytosis, and phagocytosis (Swanson, 2014; Wallroth and Haucke, 2018). During clathrin-mediated endocytosis, local synthesis of PtdIns(4,5)P_2_ from PtdIns4P by PIP kinases initiates clathrin-coated pit (CCP) formation. Subsequent conversion of PtdIns(4,5)P_2_ to PtdIns(3,4)P_2_ is necessary for CCP maturation. It has been demonstrated that elimination of PtdIns(4,5)P_2_, and generation of PtdIns(3,4)P_2_ and PtdIns3P on CCPs by PI 5-phosphatases and PI 3-kinases are the key events for maturation of endosomes (Nakatsu et al., 2010). Generation of PtdIns4P on endosomes and recruitment of PtdIns4P effectors have been reported to be necessary for recycling the plasma membrane proteins (Henmi et al., 2016). Both macropinocytosis and phagocytosis depend on actin reorganization, in which PI metabolism is known to be involved (Yeung and Grinstein, 2007; Swanson, 2014). Briefly, local accumulation of PtdIns(4,5)P_2_ stimulates actin rearrangement to form the phagocytic/macropinocytic cup. The accumulated PtdIns(4,5)P_2_ is then removed from the cup via three different mechanisms: hydrolysis by PLC to generate Ins(1,4,5)P_3_ and DAG, phosphorylation by PI 3-kinase to generate PtdIns(3,4,5)P_3_, and dephosphorylation by PI 5-phosphatases (OCRL1 and INPP5B, see below sections) to generate PtdIns4P. Removal of PtdIns(4,5)P_2_ from the cup causes actin dissociation and cup closure. On the nascent phagosomes, PtdIns3P accumulates by the action of type III PI 3-kinase and SHIP1/2 phosphatases. The generated PtdIns3P, alongside its effectors, engages in the early phase of phagosome/macropinosome maturation (Birkeland and Stenmark, 2004). In the later phase of the maturation, PtdIns3P is converted to PtdIns(3,5)P_2_, which drives sorting of cargos, such as carboxypeptidase S in yeast, and EGF receptor in human, into MVBs in cooperation with the ESCRT (endosomal sorting complex required for transport) complex (Odorizzi et al., 1998; Whitley et al., 2003). Autophagy is a mechanism necessary for bulk breakdown of cytoplasmic proteins and organelles (Mizushima et al., 2011). The unique serine/threonine kinase ULK1 (unc-51-like kinase 1, Atg1 in yeast) is activated during autophagy, and it subsequently activates the class III PI 3-kinase Vps34 complex to generate PtdIns3P on the autophagic membrane. This, in turn, recruits a variety of proteins involved in autophagosome formation (Marat and Haucke, 2016). PtdIns(3,5)P_2_ synthesis has been reported to be required at the later phase of autophagosome maturation (Ferguson et al., 2009; Zou et al., 2012; Al-Qusairi et al., 2013).

#### 2.2.3. Cytoskeletal Rearrangement, Motility, and Regulation of Transporters

As mentioned above, PtdIns4P and PtdIns(4,5)P_2_ are the major PIs on the plasma membrane, and PtdIns(3,4,5)P_3_ is transiently generated to provide a temporary signal. The importance of PtdIns(4,5)P_2_ has been well-established by a number of studies, and it has been shown that the level of PtdIns(4,5)P_2_ on the plasma membrane changes. As discussed above, PtdIns(4,5)P_2_ is involved in signal transduction and endocytosis/phagocytosis (see sections 2.2.1 and 2.2.2). PtdIns(4,5)P_2_ is also involved in the regulation of actin cytoskeleton and membrane channel activity (Balla, 2013; Hille et al., 2015; Schink et al., 2016).

During chemotaxis, chemoattractants are recognized by G-protein-coupled receptors (GPCRs) on the plasma membrane. This interaction leads to dissociation of the Gα heterodimer, which in turn activates PI 3-kinase to generate PtdIns(3,4,5)P_3_ from PtdIns(4,5)P_2_ on the cytoplasmic side of the plasma membrane. Local accumulation of PtdIns(3,4,5)P_3_ causes translocation of actin-binding proteins (ABP) that interact with PtdIns(3,4,5)P_3_, and activates actin remodeling at the leading edge of the cell. On the contrary to these events at the leading edge, the PI 3-phosphatase PTEN (phosphatase and tensin homology located on chromosome 10), which converts PtdIns(3,4,5)P_3_ to PtdIns(4.5)P_2_ to cease the signal, has been shown to accumulate at the posterior side of the cell.

There are many ion channels regulated by PIs (Hilgemann and Ball, 1996; Hille et al., 2015). Kir2.2 is a member of the inwardly rectifying potassium channel family localized on the plasma membrane, and it is known to be activated upon interaction with PtdIns(4,5)P_2_ (Hansen et al., 2011). Crystal structure analysis revealed that a direct interaction of PtdIns(4,5)P_2_ with Kir2.2 induces a structural change on this channel. This, in turn, induces the channel to compress by pulling its cytoplasmic domain toward the potassium-selective pore on the membrane, shifting the channel to the active conformation (Rohács et al., 2003; Whorton and MacKinnon, 2011). Two possible advantages of PI dependence of ion channels have been suggested: (1) to achieve local activation of the channels depending on the lipid composition (i.e., no or decreased activity during synthesis and trafficking of the lipids to the target membrane) and (2) to swiftly regulate the channel activity by lipid modifying enzymes such as PLC, PI kinases, and PI phosphatases.

#### 2.2.4. Nuclear Functions

Besides the various roles of PIs in the cytoplasm and the plasma membrane described above, PIs play indispensable roles in the nucleus. Localization of PIs, except for PtdIns(3,4)P_2_ and PtdIns(3,5)P_2_, in the nuclear matrix has been demonstrated (Payrastre et al., 1992; Vann et al., 1997; Tanaka et al., 1999; Gillooly et al., 2000; Clarke et al., 2001). Since the nuclear matrix is hydrophilic, it is not well-understood how PIs remain soluble in the nucleus (York, 2006). The significance of PtdIns(4,5)P_2_ and PtdIns5P has been well-demonstrated (Irvine, 2003; Poli et al., 2016; Hamann and Blind, 2018). PtdIns(4,5)P_2_ is involved in the transcriptional regulation and chromatin remodeling by interacting with histones (Yu et al., 1998; Cheng and Shearn, 2004; Shah et al., 2013). PtdIns(4,5)P_2_ also regulates cell cycle and differentiation through DAG generated by PLC-induced hydrolysis (Clarke et al., 2001; Newton, 2010; Poli et al., 2013, 2014). Nuclear DAG accumulation is followed by translocation of PKC to the nucleus for the phosphorylation of the target proteins (Neri et al., 1998). PtdIns5P is known to interact with TAF3, a component of the TATA box-binding protein complex, TFIID, and the chromatin-associating protein ING2, to regulate transcription and chromatin remodeling (Shi et al., 2006; Bua et al., 2013; Stijf-Bultsma et al., 2015). Interestingly, nuclear PI metabolism is regulated independently from cytoplasmic PI metabolism (Lindsay et al., 2006).

### 2.3. Spatiotemporal Regulation of PI-Mediated Signaling

#### 2.3.1. PI-Specific Binding Proteins

Spatiotemporal regulation of PI-mediated signaling occurs in a variety of biological processes by various PI-specific effectors and enzymes that mediate interconversion of PIs. The seven phosphorylated PI species are enriched on specific membrane regions in both the cytoplasm and nucleus (Balla, 2013), and specifically recognized by PI effectors. This specific recognition occurs through the interaction of the PI-specific binding domains of the effector proteins with the head groups of PIs (Figure 1). All the distinct PI-binding domains, consisting of a total of 24, have already been reported (Várnai et al., 2017).

#### 2.3.2. Major Players of PI Interconversion

Each PI interconversion reaction is regulated by specific kinases or phosphatases (Figure 1). In mammals, 18 PI interconversion reactions have been identified, and these reactions are mediated by 19 PI kinases and 28 PI phosphatases (Supplementary Tables S1, S2, Sasaki et al., 2009). PI 3-, PI 4-, and PIP kinases use PtdIns as a substrate to generate PtdIns3P, PtdIns4P, and PtdIns5P, respectively. Mono-phosphorylated PIs are further phosphorylated by PIP kinases to generate PtdIns(3,4)P_2_, PtdIns(3,5)P_2_, and PtdIns(4,5)P_2_, which is further phosphorylated to generate PtdIns(3,4,5)P_3_. Each PI is dephosphorylated by a series of PI phosphatases such as PI 3-phosphatases (PTEN, MTM) PI 4-phosphatases (INPP4, TMEM55), and PI 5-phosphatases (Synaptojanin, OCRL1, INPP5, SHIP). It has been suggested that unique expression and localization patterns of PI kinases and PI phosphatases influence the local accumulation of PIs (Balla, 2013; Schink et al., 2016).

## 3. Previous Findings on the Role of PIs in *E. histolytica*

*E. histolytica* trophozoites have been reported to have phospholipid compositions similar to those of mammalian cells except for the unique ceramide, ceramide aminoethylphosphonate (CEAP), which constitutes ~15% of the total phospholipids (Aley et al., 1980). PI is a minor phospholipid component, constituting ~5% of all phospholipids (Aley et al., 1980). Similarly, PI content of intracellular vesicles and the plasma membrane is <5%. While the plasma membrane contains less phosphatidylcoline than other membranes, it has a high content (40%) of CEAP (Aley et al., 1980). The resistance of the plasma membrane of trophozoites to the intrinsic pore forming peptide, amoebapores, is attributable to CEAP (Andrä et al., 2004).

Several previous studies demonstrated that PIs are involved in pathogenesis related processes such as adhesion, secretion, and phagocytosis. When the amebic trophozoites adhere to host cells, Gal/GalNAc lectin serves as a major adhesion molecule and transduces the signals. It is composed of heavy (Hgl), intermediate (Igl), and light (Lgl) subunits, of which Igl and Lgl are GPI-anchored. The downstream cytosolic signals transmitted from the lectin have not been well-investigated except for one example (Hughes et al., 2003). However, it has been shown that PtdIns(4,5)P_2_- and cholesterol-dependent enrichment of Gal/GalNAc lectin subunits to lipid rafts causes an increment of Ca^2+^ level followed by adhesion to the mammalian cell (Welter et al., 2011; Goldston et al., 2012). These results suggest involvement of PtdIns(4,5)P_2_-mediated Ca^2+^ signaling during cell adhesion (Goldston et al., 2012).

Cysteine proteases (CPs) are the major virulence factors. They are secreted via the default brefeldin A-sensitive or unique brefeldin A-insensitive pathways, and Rab11B-dependent pathways (Manning-Cela et al., 2003; Mitra et al., 2007). In the model organisms, it has been established that Rab11 on the secretory vesicles, and Sec3 of the exocyst complex interact, leading to tethering of the Rab11 vesicles to the plasma membrane in a PtdIns(4,5)P_2_-dependent manner (He et al., 2007; Zhang et al., 2008; Wu and Guo, 2015). The components of the exocyst complex including PtdIns(4,5)P_2_-binding Sec3 and Exo70 are mostly conserved in *E. histolytica*. Thus, it is conceivable that PI-regulated secretion takes place in *E. histolytica*.

PIs are also involved in phagocytosis in *E. histolytica* as in mammals. Several studies in which amebic transformants that expressed PI-binding proteins fused with green fluorescent protein (GFP) or in which recombinant glutathione S-transferase (GST) were used as bioprobes demonstrated that PtdIns(4,5)P_2_ were localized on the plasma membrane, while PtdIns(3,4,5)P_3_ were localized on the extended pseudopodia, and phagocytic cups, and PtdIns3P on the phagocytic cups, nascent phagosomes, and internal vesicles (Powell et al., 2006; Nakada-Tsukui et al., 2009; Byekova et al., 2010; Koushik et al., 2013). Localization of PtdIns(3,4,5)P_3_ and PtdIns3P is similar during *E. histolytica* phagocytosis and macrophage phagocytosis (Yeung and Grinstein, 2007). It has been recently shown that AGC kinases 1 and 2 that bind to PtdIns(3,4,5)P_3_ or PtdIns(3,4)P_2_ are localized to the contact site upon interaction with mammalian cells (Somlata et al., 2017). Interestingly, AGC kinases 1 and 2 have different localization patterns, although their apparent PtdIns specificities have been demonstrated with lipid overlay assay. AGC kinase 2 localizes to a tunnel-like structure proximal to the primary trogocytic cup and adjacent to the contact site on the plasma membrane during trogocytosis (“trogo” means “nibble” or “chew,” and trogocytosis is the process of internalizing live cells by nibbling them). In contrast, AGC kinase 1 is confined to the intermediate part of the trogocytic tunnel. Such an observation has been made in *E. histolytica* but not in professional phagocytes of multicellular organisms, including mammals.

Cytoskeletons also play indispensable roles during phagocytosis and trogocytosis. EhRho1, which is involved in actin rearrangement via EhFormin1 and EhProfilin1 (Bharadwaj et al., 2018), has been shown to regulate membrane blebbing to initiate internalization of the prey through PI 3-kinases (Bharadwaj et al., 2017). Inducible expression of a constitutively active EhRho1 increased the PtdIns(3,4,5)P_3_ level and reduced PtdIns(4,5)P_2_ level, whereas expression of a dominant negative EhRho1 caused opposite effects (Bharadwaj et al., 2017). EhRho1 is considered to be orthologous to HsRhoA as the amino acid sequences of their ROCK-binding domains are 65% identical. Moreover, EhRho1 complements HsRhoA activity in HEK 293T cells (Bharadwaj et al., 2017). Interestingly, HsRhoA does not localize to the phagocytic cup in mammalian cells unlike EhRho1. Signaling transduced downstream of PtdIns3P in mammals appears to be different in *E. histolytica*, because *E. histolytica* does not seem to be equipped with the orthologs of known mammalian PtdIns3P effectors. Transformation of *E. histolytica* with GFP-fused human Hrs showed that PtdIns3P was concentrated on phagosomes, more specifically at the bottom of the phagocytic cup during the early phase of phagocytosis (prior to the closure of the phagosome) (Nakada-Tsukui et al., 2009). Two PtdIns3P-binding domains are known: Phox homology (PX) and Fab-1–YGL023–Vps27–EEA1 (FYVE) domains. *E. histolytica* apparently has two PX and twelve FYVE domain-containing proteins (Nakada-Tsukui et al., 2009; N. Watanabe, data not shown). It has been demonstrated that eleven out of the twelve *E. histolytica* FYVE domain-containing proteins (EhFPs) also have a RhoGEF domain, and one of EhFP (EhFP4) preferentially binds to PtdIns4P and localizes to the plasma membrane proximal to the phagosome that is not yet closed (Nakada-Tsukui et al., 2009). Surprisingly, the C-terminal domain instead of the FYVE domain of EhFP4 binds to PtdIns3P, PtdIns4P, and PtdIns5P (Nakada-Tsukui et al., 2009). In model organisms, phagosomal PtdIns3P has been shown to recruit FYVE domain-containing proteins, which are subsequently involved in maturation of the phagosomes. However, like RhoGEF, EhFP4 appears to be primarily involved in actin rearrangement during phagocytosis eventhough full length EhFP4 does not seem to recognize PtdIns3P. Additional PtdIns3P effectors on phagosomes, most likely to be PX domain-containing proteins, still remain elusive.

## 4. PI 3-kinases

Phosphorylation of PtdIns and PIs is initially observed as conversion of PtdIns to PtdIns4P, and PtdIns4P to PtdIns(4,5)P_2_ (Balla, 2013). The enzymes responsible for these activities are named PtdIns kinases and PI kinases, respectively. Currently, it is known that some enzymes can phosphorylate both PtdIns and PIs. The enzyme classification given in this review is based on the position of the hydroxyl group that the enzymes can phosphorylate.

PI 3-kinases phosphorylate the hydroxyl group at the D3 position of the inositol ring of PtdIns, PtdIns4P, and PtdIns(4,5)P_2_ to generate PtdIns3P, PtdIns(3,4)P_2_, and PtdIns (3,4,5)P_3_, respectively. There are three subfamilies of PI 3-kinases: class I, II, and III (Sasaki et al., 2009). In general, class I enzymes preferentially generate PtdIns(3,4,5)P_3_ from PtdIns(4,5)P_2_. Class II enzymes mostly generate PtdIns(3,4)P_2_ from PtdIns4P and also generate PtdIns3P from PtdIns. Class III enzymes almost exclusively generate PtdIns3P from PtdIns. In mammals, there are a total of 8 members of PI 3-kinases. All PI 3-kinases contain a “signature motif” consisting of the catalytic kinase domain, a helical domain, also called “lipid kinase unique (LKU) domain,” and a membrane-binding C2 domain (Vanhaesebroeck et al., 2010a; Balla, 2013; Marat and Haucke, 2016). The class I to III classification of PI 3-kinases is mainly based on the presence of additional protein domains and their interactions with regulatory subunits. Class I enzymes have an adaptor binding domain (ABD), and regulatory subunit-binding and Ras binding domains (RBD). Class II enzymes have an N-terminal extension, which is involved in clathrin binding, and a C-terminal PX and extra C2 domains. It is of note that this PX domain in class II PI 3-kinase is known to preferentially bind to PtdIns(4,5)P_2_ (Stahelin et al., 2006). These domains are involved in subcellular localization and activity of the enzyme, and downstream effector selection. The class I and class III enzymes have regulatory subunits which modulate localization and activity of these enzymes.

### 4.1. Class I PI 3-Kinase

#### 4.1.1. General Description of Class I PI 3-Kinase

**Figure 3:**
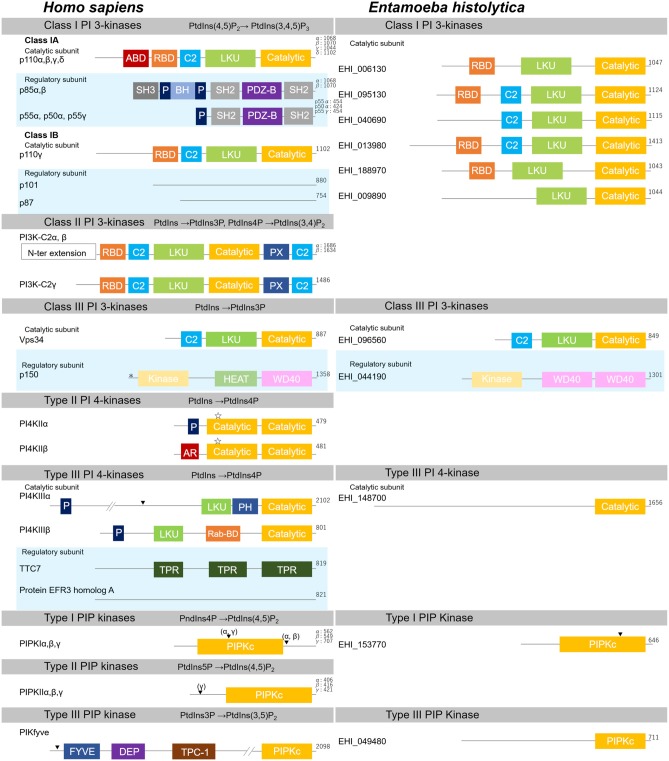
Structural features of PI kinases of *H. sapiens* and *E. histolytica*. Structural features and domain organization of PI kinases, including their regulatory subunits are shown. Numbers showing at the end of the protein indicates amino acid length. ABD, adaptor binding domain; AR, acidic region; BH, Bcl Homology; C2,C2 domain; Catalytic, lipid kinase domain of PI 3- and PI 4-kinases; DEP, disheveled, Egl-10 and pleckstrin domain; FYVE, Fab1, YOTB, Vac1, and EEA1 domain; HEAT, Huntington, Elongation factor3, PR65/A, and TOR; Kinase, Ser/Thr kinase domain; LKU, lipid kinase unique domain; P, Proline-rich; PDZ-B, PDZ domain binding domain; PH, Pleckstrin-homology; PIPKc, kinase core domain of PIP kinases; PX, Phox homology; Rab-BD, Rab binding domain; RBD, Ras binding domain; SH2, Src homology 2; SH3, Src homology 3; TPC-1, T-complex 1 homology; TPR, tetratricopeptide repeat; WD40, WD40 repeat. Myristoylation, palmitoylation sites, and the nuclear localization signal are also depicted with “*”, “
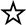
”, or “▾”, respectively.

Class I PI 3-kinases predominantly produce PtdIns(3,4,5)P_3_ from PtdIns(4,5)P_2_. There are two kinds of class I PI 3-kinases based on their composition of catalytic and regulatory subunits. One of the three class IA catalytic subunits (p110α, β, and δ) associates with one of the five p85 class regulatory subunits (p85α, p85β, p55α, p55γ, and p50α), while the class IB catalytic subunit (p110γ) associates with one of the two P101/p87 class regulatory subunits (p101 and p87) (Vadas et al., 2011; Jean and Kiger, 2014) (Figure 3). In mammals, p110α and p110β are expressed ubiquitously, while p110δ and p110γ seem to be restricted to hematopoietic cells. The class IA catalytic subunits (p110α, β, and δ) are activated via receptor tyrosine kinases and generate PtdIns(3,4,5)P_3_ at the plasma membrane. On the other hand, the p110β and p110γ catalytic subunits are activated downstream of the GPCR (Stephens et al., 1994; Stoyanov et al., 1995; Vanhaesebroeck et al., 2010a). PtdIns(3,4,5)P_3_ generation causes recruitment of PI effectors, such as the protein kinase Akt, also named protein kinase B (PKB) (James et al., 1996; Ma et al., 2008; Rodgers et al., 2017). Activated Akt is involved in cell survival and metabolism via various cellular processes, including those that involve mammalian target of rapamycin complex-1 (mTORC1), the pro-apoptotic factor BAD, and FOXO transcription factors (Vanhaesebroeck et al., 2010a; Dibble and Cantley, 2015). Due to their crucial roles in cell growth and proliferation, dominant activating mutations of the class I PI 3-kinases are known to be associated with cancers, making PI 3-kinases potential drug targets (Vanhaesebroeck et al., 2010b).

#### 4.1.2. Class I PI 3-Kinase of *E. histolytica*

In the *E. histolytica* genome (http://amoebadb.org/amoeba/), six potential catalytic subunits of class I PI 3-kinases were identified by probing the genome with the catalytic subunits of human class I PI 3-kinases as queries (NP_006209, NP_006210, AAH35683, and NP_005017, corresponding to p110α, β, γ, and δ, respectively). Independent of the query used, ten proteins were identified to share significant overall similarity, reflecting a possible redundancy among them (E-value < 1 × 10^−10^). Six of them have conserved domains, such as RBD, C2, LKU, and PI 3-kinase catalytic domains, but no protein with the ABD was identified (Figure 3; Supplementary Table S1). Four additional proteins were also identified during this survey: a class III PI 3-kinase (EHI_096560) and type III PI 4-kinase (EHI_148700) (see below), a catalytic domain-only protein (EHI_127850), and a protein that lacked the LKU domain (EHI_073560). As PI 3-kinase catalytic domain is also conserved in PI 3- and PI 4-kinases (Vogt et al., 2007), the identification of both class III PI 3-kinase and type III PI 4-kinase homologs during this search is understandable. In the present review, we tentatively designated the proteins that contain LKU and catalytic domains as class I PI 3-kinases, which excluded the four additional proteins mentioned above (Figure 3; Supplementary Table S1). Among the six class I PI 3-kinases, EHI_040690 showed a lower E-value to class II PI 3-kinase. The E-values with p110β and PI 3-kinase-C2α were 1 × 10^−107^ and 2 × 10^−110^, respectively (Supplementary Table S3 and N. Watanabe, data not shown). However, because it lacks the C-terminal domains and catalytic domain, we included this gene among the class I PI 3-kinases (also refer section 4.2.2). The six proteins with conserved RBD, C2, LKU, and PI 3-kinase catalytic domains cannot be further classified into p110α, β, γ, or δ, as none of them has the ABD and show only marginal E-value to the ABD-containing proteins p110α, β, or δ (Supplementary Table S3; Supplementary Figure S4). Furthermore, all potential class I PI 3-kinase catalytic subunit homologs showed the lowest E-value to p110β, but not for p110γ despite the fact that all the amebic homologs lack the ABD and structurally resemble p110γ (Supplementary Table S3). No homologs of the regulatory subunits that contain Src homology 2 (SH2) domain were identified in the *E. histolytica* genome database when p85α, β, p55α, p50α, and p55γ were used as the queries. Furthermore, only five proteins were predicted to have an SH2 domain and four of them were annotated as protein kinases, while the remaining protein was predicted to have a role in RNA stability and/or transcriptional regulation, with no possible link to PI 3-kinase regulatory subunits. These data suggest the possibility that the regulatory subunits of class I PI 3-kinase have been lost or replaced with a lineage-specific protein in *E. histolytica* during evolution. In *Saccharomyces cerevisiae*, class I and II PI 3-kinases are not conserved. *Dictyostelium discoideum* has catalytic but not regulatory subunits of three class I PI 3-kinases and lacks class II PI 3-kinases (Engelman et al., 2006). The catalytic subunits of *D. discoideum* class I PI 3-kinases also lack the ABD as in *E. histolytica*. Such lineage-specific modifications of the catalytic subunits and loss of the regulatory subunits of class I PI 3-kinases likely suggest divergence of PtdIns(3,4,5)P_3_-mediated lipid signaling in eukaryotes. It should be noted that *E. histolytica* has six PtdIns(3,4,5)P_3_ phosphatase homologs of PTEN, while there is only one PTEN gene in the human genome (see section 7.1).

### 4.2. Class II PI 3-Kinase

#### 4.2.1. General Description of Class II PI 3-Kinase

Class II PI 3-kinases are monomeric enzymes that generate PtdIns(3,4)P_2_ and PtdIns3P from PtdIns4P and PtdIns, respectively (Balla, 2013; Maffucci and Falasca, 2014). There are three subtypes: PI 3-kinase C2α, β, and γ, among which PI 3-kinase C2α and β have N-terminal extensions that are likely involved in autoinhibition and protein-protein interactions with clathrin (Marat and Haucke, 2016). Except for the N-terminal extensions, all class II PI 3-kinases contain one RBD, two C2, one LKU, one catalytic, and one PX domains. PI 3-kinase C2α and β isoforms are ubiquitously expressed, whereas the γ isoform is largely restricted to the liver. This class of PI 3-kinases does not have the regulatory subunit; however, they are regulated by interacting with proteins such as clathrin and Rab5 small GTPase. Clathrin associates with PI 3-kinase C2α and β isoforms through the N-terminal extension, and Rab5 interacts with the γ isoform via the RBD (Gaidarov et al., 2001, 2005; Braccini et al., 2015). Accumulating evidence suggests that class II PI 3-kinases are involved in the regulation of membrane trafficking from the plasma membrane via PtdIns(3,4)P_2_ synthesis. PI 3-kinase C2α is involved in clathrin-mediated endocytosis by the formation of PtdIns(3,4)P_2_, which constricts the membrane by recruiting PX and BAR domain-containing sorting nexin (SNX) SNX9 (Posor et al., 2013; Schöneberg et al., 2017). PI 3-kinase C2γ is recruited to endosomes as Rab5 effector for PtdIns(3,4)P_2_ synthesis, which is indispensable for delayed and sustained activation of Akt2 in the liver (Braccini et al., 2015). It was also suggested that PI 3-kinase C2α and β also play a role in the regulation of intracellular PtdIns3P levels and directly or indirectly regulate membrane traffic and autophagy (Jean et al., 2012; Devereaux et al., 2013; Franco et al., 2014).

#### 4.2.2. Class II PI 3-Kinase From *E. histolytica*

In *E. histolytica*, we concluded that there are no class II PI 3-kinases. When three human class II PI 3-kinases were used as queries, the best hits we obtained were the same proteins identified as class I PI 3-kinases (see above). As described above, because of the low similarity to class II PI 3-kinases in five out of six candidates and the absence of the PX domain in all the six, they were classified into class I PI 3-kinases. It is of note that EHI_040690 showed a lower E-value with PI3KC2α (2 × 10^−110^ with PI3KC2α and 1 × 10^−107^ with class I PI 3-kinase, p110β). Additionally, class II PI 3-kinases evolved after Metazoa, and another amoeboid organism, *D. discoideum*, lacks this class of PI 3-kinases (Engelman et al., 2006; Brown and Auger, 2011). According to these contexts, we decided to conclude that there are no class II PI 3-kinases in *E. histolytica*. However, the conservation of a gene showed low E-value with the class II PI 3-kinase, suggesting the possibility that some of the class I PI 3-kinases have a role similar to that of class II PI 3-kinases.

### 4.3. Class III PI 3-Kinase

#### 4.3.1. General Description of Class III PI 3-Kinase

The human genome has one class III PI 3-kinase, vacuolar protein sorting (Vps) 34, which phosphorylates the D3-position of PtdIns. *Vps34* gene was first identified as a temperature-sensitive mutation that impairs the sorting of vacuolar hydrolases in yeast (Herman and Emr, 1990; Schu et al., 1993). Vps34 consists of one of each of the C2, LKU, and catalytic domains, and forms a dimer with the p150 regulatory subunit (Vps15 in yeast). p150 constitutively interacts with Vps34, and the myristoyl modification in its amino terminal links Vps34 to the membrane (Stack et al., 1993; Vanhaesebroeck et al., 2010a). Since Vps34 is the only PI 3-kinase in yeast, and also widely conserved in Eukaryota, Vps34 is considered to be the ancestral PI 3-kinase (Schu et al., 1993; Engelman et al., 2006; Brown and Auger, 2011). Vps34 participate in membrane trafficking, endocytosis, phagocytosis, and autophagy through the synthesis of PtdIns3P (Sasaki et al., 2009; Swanson, 2014; Wallroth and Haucke, 2018; also see section 2.2.2). In the endocytic pathway, early endosomes mature as PtdIns3P is synthesized *in situ*, subsequently recruiting Rab5 and Rab7 to early and late endosomes, respectively. Vps34 was identified as one of the mutual effectors of Rab5 and Rab7, involved in spatiotemporal generation of PtdIns3P on endosomal membranes (Christoforidis et al., 1999; Stein et al., 2003; Shin et al., 2005).

Vps34 has been shown to form two kinds of complexes that differ in localization and function (Marat and Haucke, 2016). Complex I consists of p150, and the mammalian orthologs of yeast Vps30, Atg14, and Atg38 (Beclin-1, ATG14L, and NRBF2, respectively). It is involved in autophagy (Itakura et al., 2008; Cao et al., 2014; Lu et al., 2014). In contrast, complex II consists of p150, Beclin-1, and UVRAG, which is the mammalian ortholog of yeast Vps38. Complex II is involved in the regulation of endosome and autophagosome maturation (Kihara et al., 2001; Matsunaga et al., 2009; Funderburk et al., 2010; Sun et al., 2010; Rostislavleva et al., 2015). It is also known that, in addition to its role in autophagy Vps34 functions as an amino acid sensor, and regulates mTORC1 activity and localization (Munson et al., 2015; Hong et al., 2017). These observations suggest multiple roles of Vps34 at the cross road of nutrient sensing and membrane trafficking. Vps34 is also involved in the negative regulation of autophagy through amino acid sensing (Furuya et al., 2005; Gulati and Thomas, 2007) and mTORC1 activation mediated by PtdIns3P-dependent recruitment of phospholipase D1 (PLD1) (Yoon et al., 2011; Bridges et al., 2012). Activated mTORC1 inhibits the autophagy-promoting activity of the Complex I by phosphorylating Atg14L in the complex (Yuan et al., 2013), while it activates the Complex II by phosphorylating UVRAG. Activation of the Complex II, in turn, leads to activation of Vps34 during the reformation of lysosomes from autophagosomes following recovery from starvation (Yu et al., 2010; Munson et al., 2015; Chen and Yu, 2017). Thus, Vps34-containing complexes are interactive and involved in eliciting opposite effects in the cell.

#### 4.3.2. Class III PI 3-Kinase of *E. histolytica*

In *E. histolytica*, there are one of each Vps34 and p150 homolog (EHI_096560 and EHI_044190, respectively). Although neither their localization nor function have been demonstrated, roles of PtdIns3P are well-established as previously described (Powell et al., 2006; Nakada-Tsukui et al., 2009). During trogocytosis, which is ingestion by nibbling live mammalian cells (Ralston et al., 2014; Somlata et al., 2017), unclosed and nascent trogosomes are decorated with PtdIns3P. While localization of PtdIns3P to endosomes *per se* has not been well-documented, its localization to MVB-containing endosomes has been demonstrated (Nakada-Tsukui et al., 2009), suggesting a conserved role of PtdIns3P in the endocytic pathway in *E. histolytica*. It is conceivable that Vps34 is involved in the synthesis of PtdIns3P on trogosomes. *E. histolytica* has two TOR (EHI_155160 and EHI_169320) and two Atg8 homologs (EHI_130660 and EHI_172140). It is thus expected that *E. histolytica* Vps34 may also play a role in the response to starvation.

## 5. PI 4-kinases

Among seven PtdIns isotypes, PtdIns(4,5)P_2_ is the most abundant and well-studied in the context of PI turnover (see section 2.2.1). Since PI 4-kinase is one of the major enzymes responsible for producing the precursor of PtdIns(4,5)P_2_, it plays a significant role by producing PtdIns4P (Wang et al., 2003; D'Angelo et al., 2008). Various roles have been suggested for PtdIns4P and PI 4-kinases, including signaling on the plasma membrane (Tan and Brill, 2014). Two types of PI 4-kinases are currently known in humans: type II and type III. Type I PI 4-kinase, which was initially identified in a bovine brain homogenate chromatography fraction that showed PI kinase activity has turned out to be identical to PI 3-kinase, and thus it is no longer referred (Whitman et al., 1988). The human genome encodes two isotypes of both type II and type III PI 4-kinase. Type II and III PI 4-kinases differ in their domain structure and sensitivity to wortmannin, since the former is insensitive unlike the latter.

### 5.1. Type II PI 4-Kinase

#### 5.1.1. General Description of Type II PI 4-Kinase

Type II PI 4-kinases (PI4KII) contain a large lipid kinase domain that is separated by a long non-conserved insert. This structure is significantly different from that of type III PI 4-kinases (PI4KIII) whose catalytic domain consists of LKU and catalytic domains. It was inferred by phylogenetic analyses that type II PI 4-kinases are evolutionarily different from type III PI 4-kinases. Furthermore, type III PI 4-kinases share significant homology with the typical protein kinase PKA and PI 3-kinases (Minogue and Waugh, 2012). The catalytic domains of PI4KIIα and β are highly similar, but their N-terminal regions are divergent. The N-terminal proline-rich region (P) in PI4KIIα and acidic region (AR) in β have been shown to interact with AP-3 and AP-1 adaptor complexes, respectively (Salazar et al., 2005; Wieffer et al., 2013). Initially, PI 4-kinases were expected to have a role in the generation of PtdIns4P as a precursor of PtdIns(4,5)P_2_, whereby they were thought to regulate signal transduction from the plasma membrane. However, it has recently been suggested that type II PI 4-kinases are mostly involved in the regulation of endomembrane sorting machinery. They do so mostly in the trans-Golgi network (TGN), which functions as a sorting hub. To date, there are four suggested roles of PI4KIIα and β during membrane trafficking: (1) cargo trafficking between the TGN and internal vesicles via interaction with adaptor proteins such as AP-1 and AP-3 (Wang et al., 2003; Salazar et al., 2005; Minogue et al., 2006; Wieffer et al., 2013); (2) synthesis of PtdIns4P on mature phagosomes/autophagosomes and regulation of fusion with lysosomes (Jeschke et al., 2015; Levin et al., 2017); (3) outbound traffic toward the plasma membrane (Husebye et al., 1990; Barylko et al., 2001; Xu et al., 2006); and (4) regulation of actin-dependent trafficking by interacting with actin regulatory proteins, such as RhoGEF1, and Wiskott-Aldrich Syndrome and SCAR homolog (WASH) complex components (Mössinger et al., 2012; Ryder et al., 2013; Gokhale et al., 2016). It has been shown in mammalian cells that the two isotypes of PI4KII are differently regulated due to differences in the regulatory proteins they interact with. Both PI4KII isotypes are palmitoylated at the CCPCC motif in the catalytic domain; however, only PI4KIIβ has the ability to bind to HSP90, and the interaction is disrupted upon stimulation by epidermal and platelet-derived growth factors (Jung et al., 2011). This association with HSP90 enables stabilization of the lipid-modified PI4KIIβ in the cytosol by preventing its proteasomal degradation (Jung et al., 2011). Such elaborate mechanisms enable isotype-specific regulation of PI4KII.

#### 5.1.2. Type II PI 4-Kinase of *E. histolytica*

In *E. histolytica*, no ortholog with the E-value < 1 × 10^−4^ was identified by using human type II PI 4-kinases as a query in the BLAST search. It has been reported that a majority of parasitic protists including *Trypanosoma, Leishmania, Trichomonas vaginalis, Giardia lamblia*, and *E. histolytica*, apparently lack type II PI 4-kinases (Brown and Auger, 2011). However, apicomplexans such as *Plasmodium* exceptionally conserve a type II PI 4-kinase. Fungal and apicomplexan type II PI 4-kinase orthologs are closely related to those found in metazoans and plants, respectively. This observation is consistent with the current understanding of the evolution scheme that fungi are closely related to metazoans, and Apicomplexa acquired a plant-associated enzyme together with the plastid-like apicoplast by endosymbios (Baldauf and Palmer, 1993; McFadden, 2000). Although *Entamoeba* appears to lack type II PI 4-kinases, there is a possibility that *Entamoeba* and other organisms that lack type II PI 4-kinase may have a novel type of PI 4-kinase that is yet to be identified.

### 5.2. Type III PI 4-Kinase

#### 5.2.1. General Description of Type III PI 4-Kinase

Type III PI 4-kinases contain a continuous (uninterrupted) catalytic domain like PI 3-kinases, and both kinase types similarly show wortmannin sensitivity. Different from type II PI 4-kinases, the type III enzymes have a lipid kinase unique (LKU) domain, which is conserved among PI 3-kinases (Balla, 2013). As described above, the primary role of type III PI 4-kinases is generation of PtdIns4P, a precursor of PtdIns(4,5)P_2_, at the plasma membrane. PI4KIIIα has been shown to be recruited to the plasma membrane by interacting with two binding proteins, EFR3B and TTC7B, which are the mammalian homologs of yeast Efr3 and Ypp1 (Baird et al., 2008, see below). Additionally, knocking down PI4KIIIα causes reduction in PtdIns4P and PtdIns(4,5)P_2_ level at the plasma membrane (Nakatsu et al., 2012). Notably, in the PI4KIIIα knockout mouse embryonic fibroblast (MEF) cells, the total cellular level of PtdIns(4,5)P_2_ did not change due to the compensatory upregulation of PIPKIβ and γ, which also generate PtdIns(4,5)P_2_ from PtdIns4P. However, the level of PtdIns(4,5)P_2_ in the internal vesicles increased in the PI4KIIIα-knockout MEF cells. Several plasma membrane proteins such as M1 muscarinic receptor, and myristoylated/palmitoylated N-terminal anchor of LCK have been demonstrated to be concentrated in the internal vesicles where PtdIns(4,5)P_2_ is also enriched. These results suggest that PI4KIIIα gives unique properties to the plasma membrane, and thus lack of PI4KIIIα perturbs the membrane identity (Nakatsu et al., 2012).

Of two isotypes in humans, PI4KIIIα contains a bipartite nuclear localization sequence (NLS) and PH domain (Heilmeyer et al., 2003). In contrast, PI4KIIIβ does not have either of these domains; however, it contains several stretches rich in basic amino acids and leucine-rich sequences that can potentially serve as nuclear localization and export signals, overall suggesting their nuclear localization (Heilmeyer et al., 2003). Both PI4KIIIs have indeed been detected in the nucleus, and the yeast homolog of PI4KIIIβ, Pik1p, has been shown to shuttle between the cytosol and nucleus, suggesting its contribution to the PI pools in the nuclear speckles (Garcia-Bustos et al., 1994; de Graaf et al., 2002; Heilmeyer et al., 2003; Demmel et al., 2008; Mellman et al., 2008; Barlow et al., 2010). PI4KIIIβ plays a role as Rab11 effector, and participate in the recruitment of Rab11 to the Golgi and TGN (de Graaf et al., 2004). The crystal structures of PI4KIIIβ, Rab11, and Rab11 effector FIP3 revealed that PI4KIIIβ-Rab11 binding is independent of the kinase activity of PI4KIIIβ, which suggests a role of PI4KIIIβ other than PI phosphorylation (Burke et al., 2014). While type II PI 4-kinases are palmitoylated, type III PI 4-kinases are soluble and present in the cytosol. For membrane association, they interact with other proteins that have membrane affinity. PI4KIIIα has been shown to bind to TTC7 and EFR3 (Baird et al., 2008; Nakatsu et al., 2012). These proteins function as a scaffold for PI4KIIIα (Wu et al., 2014). For instance, EFR3 binds to acidic phospholipids, whereby it recruits the enzyme complex to the plasma membrane (Nakatsu et al., 2012). On the other hand, PIK4IIIβ binds to neuronal calcium sensor 1 (NCS-1), acyl-CoA-binding domain containing protein 3 (ACBD3), 14–3–3, and ADP-ribosylation factor 1 (Arf1) (Zhao et al., 2001; Hausser et al., 2006; Hsu et al., 2010; Sasaki et al., 2012; Klima et al., 2016). NCS-1 is a myristoylated calcium binding protein involved in membrane recruitment and activation of PI4KIIIβ. ACBD3 is a Golgi adaptor protein involved in the recruitment of PI4KIIIβ to the Golgi. Arf1 is a Golgi-localized small GTPase and its activation enhances binding and activity of PI4KIIIβ. 14–3–3 is a phosphoserine/threonine-binding protein. It binds to protein kinase D-phosphorylated PI4KIIIβ and this interaction stabilizes PI4KIIIβ activity. These binding proteins are the key regulators of type III PI 4-kinases.

#### 5.2.2. Type III PI 4-Kinase of *E. histolytica*

*Entamoeba histolytica* has only one homolog of PI4KIIIα and PI4KIIIβ (EHI_148700). As described above, *E. histolytica* does not have a type II PI 4-kinases, and EHI_148700 is the only potential PI 4-kinase in this organism. NLS search did not indicate presence of NLS on EHI_1478700 (http://nls-mapper.iab.keio.ac.jp/cgi-bin/NLS_Mapper_form.cgi) (Heilmeyer et al., 2003; Kosugi et al., 2009). Considering the analogy of *E. histolytica* to other organisms and also the fact that its type I PIP kinase is predicted to have NLS (see section 6.1.2 below), it is reasonable to speculate that *E. histolytica* also has a PtdIns4P pool in the nucleus. If so, as described above for PI4KIIIβ, basic amino acid-stretches and leucine-rich sequences in EHI_148700 may function as a nuclear localization signal. This potential *E. histolytica* type III PI 4-kinase is a soluble protein and predicted to associate with the plasma membrane through its binding proteins. However, no orthologs for the known PI4KIIIα-binding proteins TTC7 and EFR3 have been identified with an E-value lower than 1 × 10^−10^. EHI_118850 has been identified during a similarity search using human TTC7 as the query, and thus the two proteins are thought to be homologs. However, although TTC7 has three tetratricopeptide repeat (TPR) domains, EHI_118850 has two. No homologs of human/yeast ERF3 have been identified in *E. histolytica*; however, one should note that human and yeast ERF3 share only low (19.4%) amino acid homology according to Clustal Omega alignment, and no DNA similarity was detected by BLAST search. Murine homologs of EFR3 and TTC7 were identified from the PI4KIIIα immunoprecipitates of mouse brain extract (Nakatsu et al., 2012). Thus, biochemical approaches must be pursued in *E. histolytica* for the identification of its proteins that are functionally homologous to TTC7 and ERF3. It is also worth mentioning that type III PI4K of *P. falciparum* has been exploited for the development of antimalarials (McNamara et al., 2013; Kandepedu et al., 2018).

## 6. Phosphatidylinositol Phosphate Kinases (PIP Kinases)

PIP kinases have a unique catalytic domain that is not homologous to any other known lipid or protein kinases. There are three types of PIP kinases based on the substrate specificities. Type I and II PIP kinases generate PtdIns(4,5)P_2_ from PtdIns4P and PtdIns5P, respectively. Type III PIP kinases generate PtdIns(3,5)P_2_ form PtdIns3P. The only recognizable domain present in all PIPKs is the highly conserved kinase core domain (PIPKc) (Sasaki et al., 2009; Balla, 2013).

### 6.1. Type I PIP Kinase

#### 6.1.1. General Description of Type I PIP Kinase (PIP 5-Kinase)

Three type I PIP kinases (PIPKI) have been identified in humans: PIPKIα, β, and γ. The *PIPKI*γ mRNA transcript has been shown to be alternatively spliced to encode multiple forms of PIPKIγ isoforms: PIPKIγ-i1 to 6 (Ishihara et al., 1998; Giudici et al., 2004; Schill and Anderson, 2009; Xia et al., 2011). All PIPKI isoforms share a central kinase core (PIPKc) domain with 80% amino acid homology (Ishihara et al., 1998). The C-terminal part of the PIPKc domain contains a 25 amino acid activation loop that is critical for both the substrate specificity and subcellular targeting of PIPKs (Kunz et al., 2000, 2002; Liu et al., 2016). PIPKIs are the major PtdIns(4,5)P_2_-generating enzymes, which phosphorylate the hydroxyl group at the D5 position of the inositol ring of PtdIns4P, and have a wide variety of roles relating to PtdIns(4,5)P_2_ synthesis (Rameh et al., 1997). Since one of the major roles of PtdIns(4,5)P_2_ is the actin-mediated processes, PIPKIs are also indispensable for the actin dynamics. In fact, yeast has a single PIPKI, Mss4p, and the *mss4* mutant showed a phenotype similar to actin deficiency (Desrivières et al., 1998; Homma et al., 1998). In mammals, PIPKIs have also been shown to be involved in actin dynamics and membrane activities by generating PtdIns(4,5)P_2_ from PtdIns4P, and the specific role of each PIPK seems to vary depending on their expression levels and the cell type (Balla, 2013). PIPKIs are widely distributed in the cell, and each isoform shows a unique localization pattern, whereby it regulates a specialized (compartmentalized) pool of PtdIns(4,5)P_2_ (Doughman et al., 2003; Tan et al., 2015). PIPKIγi1–3 and 5 have been shown to be localized on the plasma membrane (Balla, 2013), while PIPKIγi2 is also localized in the recycling endosomes and focal adhesions (Di Paolo et al., 2002; Ling et al., 2002). It has been also shown that PIPKIα and β are targeted to autolysosomes (Rong et al., 2012). Additionally, it has been independently demonstrated that PIPKIα and PIPKIγi4 are found in nuclear speckles (Li et al., 2013), and PIPKIβ accumulates at the perinuclear regions (Doughman et al., 2003). PIPKIs apparently play redundant roles, and only a single copy of PIPKIγ is sufficient to support the development and growth of mice to the adulthood (Volpicelli-Daley et al., 2010). All three PIPKI isozymes have been linked to endosomal traffic (Galiano et al., 2002; Shinozaki-Narikawa et al., 2006). PIPKIα and β are known to initiate lysosomal reformation during autophagy (Yu et al., 2010; Rong et al., 2012; Chen and Yu, 2017). PIPKIα and γ are also implicated in chemotaxis (Lacalle et al., 2007; Lokuta et al., 2007). PIPKIγi1 is involved in the generation of pools of PtdIns(4,5)P_2_ for Ins(1,4,5)P_3_, which regulates calcium release in histamine-stimulated HeLa cells (Wang et al., 2004). PIPKIα has been shown to be involved in pre-mRNA processing in association with non-canonical poly(A) polymerase, Star-PAP, which is specifically stimulated by PtdIns(4,5)P_2_ (Mellman et al., 2008).

Activation of PIPKI differs from that of type II PIP kinase (PIPKII). PIPKI activity is stimulated by phosphatidic acid (PA) (Jenkins et al., 1994). Phospholipase D (PLD) and diacylglycerol kinase produce PA and are thought to be involved in the activation of PIPKIs (Tolias et al., 1998; Honda et al., 1999; Divecha et al., 2000). In mammals, two PLD isotypes use PtdIns(4,5)P_2_ as a cofactor (Cockcroft, 2001), and thus, locally accumulated PIPKI and PLD mutually activate each other through their products, which results in a positive feedback loop (Mahankali et al., 2015). It has been shown that Arf6, a small GTPase which regulates membrane traffic, recruits PLD2 to membrane ruffles and stimulates PIPKI activity (Skippen et al., 2002). PLD1 is involved in initiation of autophagy by stimulating the PIPKI activity to generate necessary PtdIns(4,5)P_2_ pool for the formation of the isolation membrane (Jenkins and Frohman, 2005; Dall'Armi et al., 2010; Fan et al., 2011; He et al., 2013).

#### 6.1.2. Type I PIP Kinase (PIP 5-Kinase) of *E. histolytica*

In *E. histolytica*, only one possible ortholog (EHI_153770) with the E-value of < 1 × 10^−10^ was found (E-value, 1 × 10^−38^) by using three *H. sapiens* type I PIP kinases (PIPKI) (NP_001129110, AAH30587, and NP_001287778) as queries. EHI_153770 has a PIPKc domain based on pfam search. Furthermore, human and yeast PIPKIs, PIPKIβ (NP_001265182) and Mss4p (BAA02869), respectively, were identified with the corresponding E-values of 8 × 10^−39^ and 5 × 10^−37^, when EHI_153770 was used as a query to search for a human or yeast ortholog, respectively. Since no PIPKII ortholog has been identified in *E. histolytica* (see below section 6.2.2), EHI_153770 likely has a major role in PtdIns(4,5)P_2_ generation from PtdIns4P. This single type I PIP kinase should have a wide variety of roles in *E. histolytica*. It is of note that the NLS is conserved in EHI_153770 and the putative PLD of *E. histolytica* also has an NLS (K. Das, data not shown). Sharma and colleagues recentrly demonstrated of EHI_15377 (Sharma et al., 2019).

### 6.2. Type II PIP Kinase

#### 6.2.1. General Description of Type II PIP Kinase (PIP 4-Kinase)

Type II PIP kinase is the oldest PIP kinase identified among the others. However, the role of this class of enzymes is not as well-understood as that of type I PIP kinases (Boronenkov and Anderson, 1995; Divecha et al., 1995). Although PIPKII was initially thought to be responsible for generation of PtdIns(4,5)P_2_ from PtdIns5P, this enzyme is currently considered to play a role in the regulation of the PtdIns5P levels (Clarke et al., 2010). Three PIPKII isotypes, PIPKIIα, β, and γ, are known, all of which contain the conserved PIPKc kinase domain bisected in the center by a non-conserved inserted sequence (Loijens et al., 1996; Itoh et al., 1998; Sasaki et al., 2009). All three PIPKIIs contain the ~25 amino acid activation loop at the C-terminal part of the PIPKc domain as in PIPKIs (Sasaki et al., 2009). PIPKII α, β, and γ isotypes differ in their relative enzymatic activities in the order listed, with α being the most active (Clarke et al., 2008; Bultsma et al., 2010). It was speculated that the weak forms of PIPKII (β and γ) dimerize with the strong enzyme PIPKIIα and serve as adaptor proteins that bring it to specific membrane compartments (Clarke et al., 2010). Nuclear localization of PIPKIIα and PIPKIIβ has been reported (Bultsma et al., 2010; Wang et al., 2010). PIPKIIβ, which lacks an NLS, is targeted to the nucleus by a unique nuclear localization sequence consisting of an acidic α helix present in its unique insertion region in the kinase domain (Ciruela et al., 2000; Bunce et al., 2008). In contrast, PIPKIIγ has been detected in the ER by immunochemistry and subcellular fractionation, and also in other compartments of the endomembrane system (Itoh et al., 1998; Clarke et al., 2009).

Type II PIP kinase does not seem to play a role in the regulation of actin dynamics, as human type II PIP kinase failed to rescue yeast mss4 (type I PIP kinase) deficiency (Homma et al., 1998; Ishihara et al., 1998). It has been shown that PIPKIIα is involved in the formation and secretion of alpha granules in platelets (Rozenvayn and Flaumenhaft, 2001, 2003; Schulze et al., 2006). PIPKIIβ knockout mice show increased insulin sensitivity, likely through enhanced Akt activity (Carricaburu et al., 2003; Lamia et al., 2004). This is tentatively explained by slow degradation of PtdIns(3,4,5)P_3_ in the knockout mice; however, the molecular basis remains elusive. The hypothesis that an excess amount of PtdIns5P inhibits phosphatase activity has been rejected (Campbell et al., 2003; Schaletzky et al., 2003). It has been shown that increasing the PtdIns5P level by overexpressing a bacterial PtdIns(4,5)P_2_4-phosphatase (IpgD) enhanced Akt activity (Pendaries et al., 2006). It has also been shown that nuclear PIPKIIβ is involved in nuclear stress response. For instance, UV irradiation induces phosphorylation of Ser236 in PIPKIIβ, and this phosphorylation inactivates PIPKIIα kinase-activity, which is associated with PIPKIIβ and accumulation of PtdIns5P (Jones et al., 2006; Bultsma et al., 2010; Wang et al., 2010) (also see section 2.2.4).

#### 6.2.2. Type II PIP Kinase (PIP 4-Kinase) of *E. histolytica*

The *E. histolytica* genome was found to encode one possible PIPKI with a reasonable E-value of 6 × 10^−26^ when three human PIPKII isotypes were used as queries in BLAST search. This protein (EHI_153770) was identified as a top hit with a lower E-value of 6 × 10^−37^ when PIPKI was used as a query, as described above. Because of this higher similarity to PIPKIs, EHI_153770 is categorized as a PIPKI.

### 6.3. Type III PIP Kinase

#### 6.3.1. General Description of Type III PIP Kinase (PIP 5-Kinase)

**Figure 4:**
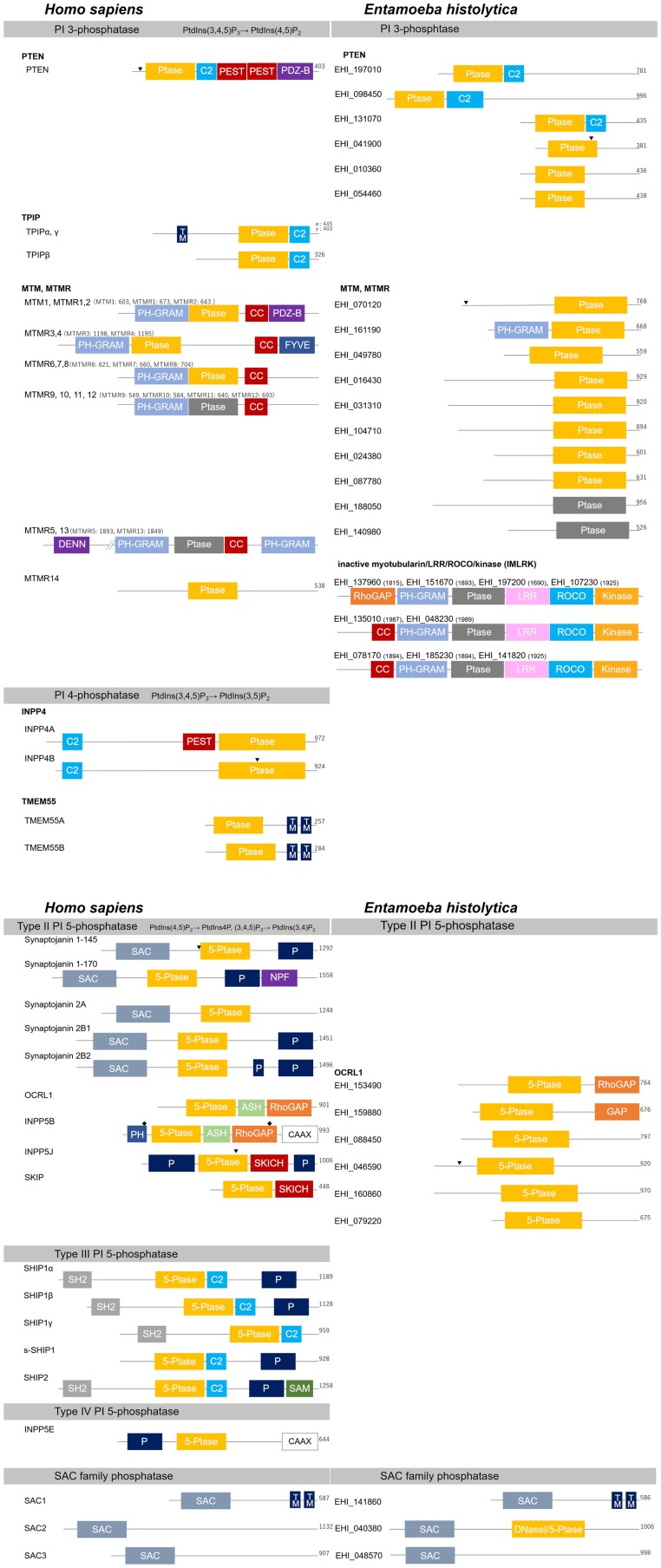
Structural features of PI phosphatases of *H. sapiens* and *E. histolytica*. Structural features and domain organization of the PI phosphatases are shown. Numbers showing at the end of the protein or after the name of the protein indicates amino acid length. 5-Ptase, PI 5-phosphatase domain; ASH, ASPM-SPD2-hydin; C2, C2 domain; CAAX, CAAX motif; CC, coiled coil domain; DENN, differentially expressed in normal vs. neoplastic; DNaseI, DNase I-like domain; FYVE, Fab1, YOTB, Vac1, and EEA1 domain; GRAM, glucosyltransferases Rab-like GTPase activators and myotubularins; GAP, GTPase-activating domain; kinase, protein kinase domain; LRR, leucine-rich repeats; NPF, asparagine- proline-phenylalanine repeats; P, Proline-rich; PDZ-B, PDZ domain binding domain; PEST, Proline, glutamine, serine, threonine; PH, Pleckstrin-homology; (Ptase; gray), inactive Ptase domain; (Ptase; yellow), active CX5R motif containing PI 3- or PI 4-phosphatase domain; ROCO, comprised of a ROC (Ras of complex proteins) and COR (C-terminal of ROC) region; SAC, Sac domain; SAM, sterile alpha motif; SH2, Src homology 2; SKICH, SKIP carboxylhomology; TM, transmembrane domain. Clathrin-binding domain and the nuclear localization signal are also labeled with “♦” and “▾”, respectively.

Type III PIP kinases (PIPKIII) phosphorylate PtdIns3P to PtdIns(3,5)P_2_, which is one of the least abundant PIs (Hasegawa et al., 2017). PIPKIII was initially found in yeast by a genetic screening for defects in nuclear segregation (Yamamoto et al., 1995). A PIPKIII-deficient yeast line showed enlargement of vacuoles and retardation in vacuole delivery of hydrolases, such as carboxypeptidase Y (CPY) (Gary et al., 1998). This observation suggests the primary effect of PIPKIII deficiency is on membrane trafficking. PIPKIII is a large protein of >2000 amino acids and contains the C-terminal catalytic domain of PIPKc, which is similar to that of PIPKI and PIPKII. Distinct from other PIP kinases, PIPKIIIs have multiple domains in humans: FYVE (Fab1p, YOTB, Vac1p, and EEA1), DEP (disheveled, Egl-10, and pleckstrin), and TCP-1 (t-complex polypeptide-1) domains (Sasaki et al., 2009; Balla, 2013). These domains are involved in PtdIns3P binding, membrane association, and actin/tubulin binding, respectively (Cabezas et al., 2006). As PtdIns(3,5)P_2_ has a critical role in endosome/lysosome biogenesis, PtdIns3P, which is highly used in endocytic pathways, is converted to PtdIns(3,5)P_2_ by PIPKIII on endosomes to initiate MVB formation (Odorizzi et al., 1998). PIPKIII deficiency in mammalian cells has also been shown to cause massive vacuolization (enlarged endosomes) due to defective MVB formation (Ikonomov et al., 2003). PIPKIIIs are involved in a variety of membrane traffic pathways and signaling, such as retrograde transport of cation-independent mannose 6-phosphate receptor and sortilin, lysosomal localization and activity of mTORC1, and autophagosome-lysosome fusion (Rutherford et al., 2006; Zhang et al., 2007; Bridges et al., 2012; Hasegawa et al., 2016; Jin et al., 2016). It is of note that suppression of PIPKIII hampered phagosome maturation but did not inhibit acidification of lysosomes in macrophages (Kim et al., 2014). Additionally, PIPKIIIs are involved in nutrient (or macromolecule) import via vacuoles (Krishna et al., 2016). PIPKIII thus plays multiple roles by tightly regulating PtdIns3P and PtdIns(3,5)P_2_ concentrations in vesicular trafficking. In mammals and yeast, PIPKIIIs are known to form a complex with the Sac1-related PI phosphatase, Fig4/Sac3, and a scaffold protein, Vac14/ArPIKfyve. In yeast, PIPKIII is also known to interact with the WD domain protein Atg18 and Vac7 (Duex et al., 2006; Chow et al., 2007; Sbrissa et al., 2007; Botelho et al., 2008; Jin et al., 2008). Deletion of Fig4/Sac3 and Vac14/ArPIKfyve reduces PtdIns(3,5)P_2_ level (Gary et al., 1998, 2002; Bonangelino et al., 2002; Dove et al., 2002; Rudge et al., 2004; Duex et al., 2006; Zhang et al., 2007; Zolov et al., 2012). Thus, PIPKIII activation and stabilization is also regulated by the associated proteins, such as phosphatases and scaffold proteins in the complex, to tightly control the PtdIns(3,5)P_2_ level.

#### 6.3.2. Type III PIP Kinase of *E. histolytica*

A genome survey of *E. histolytica* by a BLAST search with *H. sapiens* PIPKIII as the query identified one ortholog candidate (EHI_049480). EHI_049480 shows similarities to human PIPKI and PIPKIII with the E-values of 5 × 10^−4^ and 4 × 10^−45^, respectively. EHI_049480 is considerably smaller than the potential human homolog, and has not been predicted to contain any additional domains such as FYVE. However, based on the highest similarity between the catalytic domains of EHI_049480 and human PIPKIII, we tentatively annotated EHI_049480 as a *E. histolytica* PIPKIII. Additionally, a BLASTP search against the *H. sapiens* and *S. cerevisiae* genomes with EHI_049480 as the query identified PIPKIII and Fab1 with the E-values of 2 × 10^−45^ and 9 × 10^−40^, respectively.

We further searched for other PIPKIII complex components such as Vac7, Vac14, Atg18, and Fig4 (the yeast homolog of the mammalian Sac3). Potential orthologs for Atg18 and Fig4 were identified with the E-values of 1 × 10^−20^ and 1 × 10^−34^, respectively. This possible Fig4 ortholog showed a lower E-value with Sac1 (1 × 10^−82^). Thus, it is reasonable to tentatively assign this protein as a Sac1 ortholog, although it is not possible to specifically categorize Sac1 among Sac orthologs (see section 10). A human ortholog of Atg18 (WIPI), which is a WD repeat-containing protein that interacts with phosphoinositides, recognizes PtdIns3P on the nascent autophagosome and recruits the lipidation machinery to the autophagosome for LC3. However, the role of WIPI in PtdIns(3,5)P_2_ metabolism remains unknown. It is unknown whether *E. histolytica* has PtdIns(3,5)P_2_ metabolic pathways similar to other organisms. However, conservation of ESCRT and MVB, and the localization of PtdIns3P on phagosomes indicate that PtdIn(3,5)P_2_-mediated vesicular trafficking is also conserved in *E. histolytica*. We failed to identify potential Vac7 and Vac14 homologs with an E-value < 1 × 10^−1^. As 19 HEAT repeat-containing proteins are present in the *E. histolytica* genome, it is possible that some of them function in lieu of Vac14.

## 7. PI 3-Phosphatases

PI phosphatases are also important regulators of PI signaling. Because identification of lipid kinases and PLC-mediated second messengers had a significant impact, studies on phosphatase activity in the early '80s was largely focused on Ins(1,4,5)P_3_ decomposition. A number of inositol phosphatases and PI phosphatases were identified and characterized (Majerus et al., 1986). In the ‘90s, it was revealed that mutations in PI phosphatases are responsible for human genetic disorders, including Oculo-Cerebro-Renal Syndrome of Lowe (OCRL), human X-linked centromyotubular myopathy, and Charcot-Marie-Tooth disease type 4B. OCRL1, myotubularin-related (MTMR) 1, and MTMR2 were identified as the genes responsible for the above-mentioned human genetic disorders, respectively (Attree et al., 1992; Myers et al., 1997; Maehama and Dixon, 1998, 1999; Blondeau et al., 2000; Taylor et al., 2000; Kim S. A. et al., 2002). One of the most studied PI phosphatases, PTEN, was identified as a tumor suppressor gene (Maehama and Dixon, 1998). Similar to PI kinases, classification of PI phosphatases is primarily based on the position of the hydroxyl group that they dephosphorylate. The human and yeast genomes are known to encode twenty-eight and six PI phosphatases, respectively (Odorizzi et al., 2000; Sasaki et al., 2009).

PI 3-phosphatases are categorized into two groups based on substrate specificities. One group includes PTEN, TPTE (transmembrane phosphatase with tensin homology), and TPIP (TPTE and PTEN homologs inositol lipid phosphatase), while the other group includes myotubularins (MTMs). Since TPTEs have no phosphatase activity, and their functional role remains unclear, they are not discussed in this review. However, TPTE has been reported to be associated with cancers. For instance, it has been demonstrated that TPTE is upregulated in prostate cancer, and autoantibody production against TPTE is observed in lung cancer (Walker et al., 2001; Tapparel et al., 2003; Bansal et al., 2015; Kuemmel et al., 2015). PTEN and TPIP have similar catalytic domains but differ in substrate specificity. PTEN removes the phosphate moiety at D3 position of PtdIns(3,4,5)P_3_ and PtdIns(3,4)P_2_, while TPIPs dephosphorylate any of the 3′-phosphorylated inositides at D3 position (Walker et al., 2001; Malek et al., 2017). On the other hand, MTMs remove the D3 phosphate from PtdIns(3,5)P_2_ and PtdIns3P. The catalytic center of both groups of PI 3-phosphatases contains the CX_5_R motif, which is also found in protein tyrosine phosphatases (Hsu and Mao, 2015).

### 7.1. PTEN and TPIP

#### 7.1.1. General Descriptions of PTEN and TPIP

PTEN was initially identified as a tumor suppressor gene located on chromosome 10 (Li et al., 1997; Steck et al., 1997; Maehama and Dixon, 1998). It is among the most frequently mutated genes in various cancers in humans (Guldberg et al., 1997; Li et al., 1997; Steck et al., 1997; Tashiro et al., 1997; Cairns et al., 1998; Kohno et al., 1998; Maehama, 2007), and in hereditary cancer predisposition syndromes, such as Cowden disease (Myers et al., 1997; Furnari et al., 1998). Human TPIP was bioinformatically identified as a protein encoded by PTEN-related genes (Walker et al., 2001). PTEN consists of a protein tyrosine phosphatase (PTP)-related lipid phosphatase domain, a C2 domain, two PEST (proline, glutamine, serine, threonine) sequences, and a PDZ domain (Figure 4). The C2 domain is known to be involved in lipid binding and protein stability. PEST sequences are known to enhance proteolytic sensitivity, and the PDZ domain is involved in protein-protein interactions (Maehama, 2007; Sasaki et al., 2009; Balla, 2013). The human genome encodes three isotypes of TPIP, and only two of them have an N-terminal transmembrane domain (Figure 4). PTEN and TPIP have a CX_5_R motif-containing PTP-related lipid phosphatase domain, whose core sequence is CKAGKGR and CKGGKGR, respectively. This domain forms the catalytic cleft of PTEN, and it is wider than the corresponding domain of protein tyrosine phosphatases. This allows the bulky PtdIns(3,4,5)P_3_ head group to access the active center of the enzyme (Lee et al., 1999). The principal role of PTEN is to cease the cell proliferation signal by inactivating Akt though dephosphorylation of PtdIns(3,4,5)P_3_, whereby it serves as a tumor suppressor.

PTEN is predominantly localized to the cytosol, and also dynamically associated with the plasma membrane, where it hydrolyzes PtdIns(3,4,5)P_3_ (Billcliff and Lowe, 2014). Although PTEN lacks an NLS, it also localizes to the nucleus and plays important roles in chromosome stability by directly interacting with centromere specific binding protein C (CENP-C), DNA repair by interacting with p53 and Rad51, and cell cycle regulation by interacting with APC and MAP kinases (Freeman et al., 2003; Chung and Eng, 2005; Chung et al., 2006; Tang and Eng, 2006a,b; Shen et al., 2007; Trotman et al., 2007; Song et al., 2011). This nuclear translocation depends on the cytoplasmic localization signal and ubiquitination (Denning et al., 2007; Trotman et al., 2007; Wang et al., 2007; Drinjakovic et al., 2010). Mutations in the N-terminal cytoplasmic localization signal, which is found in familial Cowden disease patients, increases nuclear localization (Denning et al., 2007). Mono-ubiquitination of K13 and K298 serves as the nuclear translocation signal, while poly-ubiquitination causes degradation of PTEN (Trotman et al., 2007; Wang et al., 2007; Drinjakovic et al., 2010). PTEN has also been shown to participate in phagocytosis, autophagy and determination of cell polarity through dephosphorylation of PtdIns(3,4,5)P_3_ (Arico et al., 2001; Kim J.S et al., 2002; Martin-Belmonte et al., 2007). In contrast, the physiological role of TPIP is not well-understood. TPIPα and γ both have the transmembrane domain unlike TPIPβ. Accordingly, they are localized on internal membranes, whereas TPIPβ remains in the cytosol (Walker et al., 2001).

#### 7.1.2. PTEN and TPIP of *E. histolytica*

The *E. histolytica* genome potentially encodes six PTEN orthologs with E-values < 1 × 10^−10^ to the human PTEN (Figure 4; Supplementary Table S2; Supplementary Figure S5). Domain search by pfam showed that these six PTEN orthologs contain the CX_5_R motif-containing phosphatase domain. Furthermore, the same protein candidates were detected when the three human TPIP isoforms were used as queries. The E-values against TPIP were around 1 × 10^−16^-10^−17^, which is higher than those against PTEN (Supplementary Table S2). Thus, we concluded that they are likely PTEN orthologs. Furthermore, five out of the six orthologs have a CX_5_R motif identical to that found in PTEN (CKAGKGR). The CX_5_R sequence of the remaining ortholog (EHI_131070) is CLAGRGR. Three of the *E. histolytica* PTEN orthologs also had a C2 domain. PTENs commonly have the cytosol localization signal (Supplementary Figure S2) (Denning et al., 2007). Among the three C2 domain-containing candidates, all six consensus amino acids of the cytosol localization signal are conserved in EHI_131070, and all but one amino acid are also conserved in EHI_197010 and EHI_098450 (Supplementary Figure S2). In the three *E. histolytica* PTEN candidates, which lack the C2 domain, only a few residues are conserved (four in EHI_010360, EHI_054460; three in EHI_041900) (Supplementary Figure S2). All the three candidates have lysine or tyrosine instead of phenylalanine that is found in the amino acid 22 position of human PTEN. It has been shown that F21A mutation causes nuclear localization of PTEN, which in turn fails to activate Akt even though the phosphatase activity is retained (Denning et al., 2007). Thus, it is not clear if the three *E. histolytica* PTEN candidates which lack the C2 domain have functional cytosol localization signals. According to the gene expression profile, two of the PTEN homologs, EHI_197010 and EHI_098450, are actively transcribed (Supplementary Figure S1). Both of them have a C2 domain and the cytosol localization signal.

### 7.2. Myotubularin (MTM)

#### 7.2.1. General Description of MTM

The human myotubularin (MTM) family consists of 15 members [MTM1 and MTM related (MTMR) 1–14]. As in PTEN, the CX_5_R motif of their phosphatase domain is overall well-conserved. Among the 15 MTMs, six of them (MTMR5 and MTMR9–13) substituted the conserved cysteine and arginine residues within the CX_5_R motif with other amino acids, and thus these MTMs are catalytically inactive (Laporte et al., 2003; Hnia et al., 2012; Hsu and Mao, 2015). The CX_5_R motif in MTMs (CXXGWDR) is slightly different from that in PTEN (CKAGKGR). While PTEN prefers PtdIns(3,4,5)P_3_ and PtdIns(3,4)P_2_ as substrates, MTMs preferentially dephosphorylate PtdIns3P and PtdIns(3,5)P_2_. MTMs are categorized into six groups based on their domain configurations and catalytic activity: MTM1 and MTMR1–2; MTMR3–4; MTMR6–8; MTMR14; MTMR5 and 13; and MTMR9–12 (Figure 4). In all MTMs, except for MTMR14, the PH-GRAM (pleckstrin homology-glucosyltransferase, Rab-like GTPase activator and myotubularin) domain is conserved. This domain is involved in PI binding. Additionally, they all have an active or inactive catalytic core, and coiled-coil domain, which is involved in homo- or hetero- dimerization. In addition, some members have the C-terminal PDZ binding sequence and FYVE domain. The N-terminal DENN and C-terminal PH domains are conserved in two of the catalytically inactive MTM members, MTMR5 and MTMR13. Although all the catalytically inactive members (MTMR5 and MTMR9–13) lack an active phosphatase domain, they can heterodimerize with active MTMs, whereby inactive MTMs likely regulate the activity and localization of active MTMs (Kim et al., 2003; Mochizuki and Majerus, 2003; Lorenzo et al., 2006). The role of MTMs and their preferred substrates [PtdIns3P and PtdIns(3,5)P_2_] in endocytosis and membrane traffic have been well-characterized (Robinson and Dixon, 2006; Hohendahl et al., 2016). Besides these, MTMs have been suggested to have other roles in cellular processes, including cell proliferation and differentiation, autophagy, phagocytosis, organelle positioning, cytokinesis, cytoskeletal rearrangement, and cell junction dynamics (Hnia et al., 2012; Lawlor et al., 2016). It is of note that some of the cellular functions performed by MTMs depend on the tissue-specific expression patterns of their binding proteins and do not involve a phosphatase activity. For instance, disrupting the interaction between MTM1 and its intermediate filament, desmin, causes the formation of desmin aggregates, and this impairment is associated with myofibrillar myopathies and cardiomyopathies (Hnia et al., 2011).

#### 7.2.2. MTM of *E. histolytica*

Genome-wide survey against the *E. histolytica* genome using human MTM1, MTMR3, 5, 6, 9, or 14 as the query identified an identical set of 11 proteins with E-values < 1 × 10^−58^. We concluded that 10 out of these 11 potential homologs are *E. histolytica* MTM orthologs by pfam search, because they contain a myotubularin-like phosphatase domain (Figure 4; Supplementary Tables S1, S4). All of them, except EHI_140980 and EHI_188050, contain the conserved catalytic domain. The phosphatase domains of these two exceptions conserved the cysteine residue in their C(S/T)DGWDR motifs, but arginine is replaced with serine or isoleucine (CRNGWDS and CIDGTGI, respectively). Although *E. histolytica* MTMs appear to have a simpler domain organization, they also are thought to heterodimerize as in model organisms given that the genome contains both active and inactive MTMs. Among all the PI phosphatases in *E. histolytica*, MTMs are the most diverged ones.

It should be noted that Amoebozoa supergroup members exclusively have a protein family that contain multiple inactive myotubularin domains. These proteins have been designated as inactive myotubularin/LRR/ROCO/kinase (IMLRK) proteins (Kerk and Moorhead, 2010). Nine IMLRK proteins have previously been identified in the *E. histolytica* genome by the FFAS03 (Fold and Function Assignment System) sequence: profile method and the HHPred [Hidden Markov Model (HMM)-HMM structure prediction] profile: profile method (Kerk and Moorhead, 2010). The *D. discoideum* homologs of IMLRKs, Pats1 and GbpC, have been identified as a cytokinesis-related protein and cGMP-binding protein, respectively (Goldberg et al., 2002; Abysalh et al., 2003). Since both *D. discoideum* IMLRK homologs are involved in cytoskeleton-related processes such as cytokinesis and chemotaxis, *E. histolytica* IMLRKs may also be involved in the similar processes (Bosgraaf et al., 2002, 2005; Abysalh et al., 2003; Lewis, 2009). However, it is not clear why *E. histolytica* has inactive myotubularin domains, and more IMLRK proteins than *D. discoideum* (nine vs. two, respectively).

## 8. PI 4-Phosphatases

Dissimilar to PI 3- and PI 5-phosphatases, which belong to multiple families of enzymes, PI 4-phosphatases consist of only four proteins. All members of human PI 4-phosphatases have the CX_5_R motif in the catalytic domain. There are two groups of PI 4-phosphatases: inositol polyphosphate-4-phosphatase (INPP4) and transmembrane protein 55 (TMEM55) (Figure 4). INPP4 dephosphorylates PtdIns(3,4)P_2_, and TMEM55 dephosphorylates PtdIns(4,5)P_2_. No PI 4-phosphatases that use PtdIns(3,4,5)P_3_ as a substrate have been identified yet. *E. histolytica* has no homologs of PI 4-phosphatases in this class. Here, we will discuss only the general aspects of PI 4-phosphatases.

### 8.1. INPP4

Two INPP4 proteins have been identified in the human genome and named INPP4A and B. They both contain a conserved catalytic domain and N-terminal C2 domain; however only INPP4A contains the PEST sequence (Sasaki et al., 2009; Balla, 2013) (Figure 4). The C2 domains of these proteins show different binding specificities. The C2 domain of INPP4A preferentially binds to PtdIns(3,4)P_2_, PtdIns3P, phosphatidylserine, and calcium (Ivetac et al., 2005, 2009; Shearn and Norris, 2007), while that of INPP4B prefers phosphatidic acid and PtdIns(3,4,5)P_3_ (Ferron and Vacher, 2006). INPP4A is cleaved and inactivated by calpain via recognition of the PEST sequence in INPP4A (Norris et al., 1995), as shown in platelets stimulated with thrombin and calcium ionophores (Norris et al., 1997). INPP4A is known to be localized in recycling and early endosomes in resting cells and translocated to the plasma membrane upon serum stimulation (Ivetac et al., 2005). In contrast, INPP4B has been shown to be localized diffusely in the cytoplasm. INPP4A is involved in the regulation of membrane traffic, which is consistent with its endosomal localization. It has been shown that INPP4A is activated by Rab5, and knocking down Rab5 inhibits transferrin uptake (Shin et al., 2005). On the other hand, overexpression of INPP4A suppresses the enlarged endosome morphology caused by PtdIns3P deficiency in INPP4A knock-out MEF cells (Ivetac et al., 2009), suggesting that INPP4A produces PtdIns3P from PtdIns(3,4)P_2_. A significant role of INPP4A in the generation of PtdIns3P to recruit SNX9 and actin polymerization machineries during clathrin-mediated endocytosis has also been reported (Malek et al., 2017). No potential orthologous genes with the E-value < 1 × 10^−1^ have been identified in *E. histolytica*.

### 8.2. TMEM55

TMEM55 (transmembrane protein 55), which was named after the transmembrane regions it contains, was originally identified based on its homology to the virulence factor of *Burkholderia pseudomallei*, BopB, a putative phosphatase that contains a CX_5_R motif (Ungewickell et al., 2005). There are two isotypes of TMEM55, termed TMEM55A and B. They both consist of a CX_5_R motif-containing phosphatase domain and two putative transmembrane domains at the C-terminus (Rynkiewicz et al., 2012). Based on *in vitro* and *in vivo* observations, TMEM55 specifically hydrolyzes the D4 phosphate of PtdIns(4,5)P_2_. Both TMEM55A and TMEM55B show cytosolic and late endosomal membrane localization (Ungewickell et al., 2005). Overexpression of TMEM55A enhances EGFR degradation induced by EGF stimulation, suggesting that TMEM55A is involved in the endocytic and recycling pathways (Ungewickell et al., 2005). Additionally, TMEM55A has been reported to be involved in macrophage phagocytosis (Morioka et al., 2018). On the other hand, TMEM55B translocates from the cytosol to the nucleus and increases the PtdIns5P level in response to DNA damage (Zou et al., 2007). This upregulation of PtdIns5P in the nucleus is accompanied by activation of ING2 (Gozani et al., 2003) and enhanced p53-mediated cell death (Zou et al., 2007). In *E. histolytica*, no potential orthologous genes with the E-value < 1 × 10^−1^ have been identified. It is possible that *E. histolytica* utilizes Sac phosphatase (see below) for dephosphorylation of the D4 position of PIs.

## 9. PI 5-Phosphatases

Inositol polyphosphate 5-phosphatases (INPP5s) have an inositol 5-phosphatase (5-Ptase) domain that contains two signature motifs (F/Y)WXGDXN(F/Y)R and P(A/S)(W/Y)(C/T)DR(I/V)L(W/Y) separated by ~60–75 residues (Majerus et al., 1999). INPP5s are Mg^2+^-dependent enzymes and share homology with apurinic/apyrimidinic family of endonucleases (Whisstock et al., 2000). There are four classes of PI 5-phosphatases (types I–IV). However, the type I enzyme (INPP5A) does not have lipid phosphatase activity, and dephosphorylate Ins(1,4,5)P_3_ and Ins(1,3,4,5)P_4_ (Laxminarayan et al., 1993, 1994; De Smedt et al., 1994). Type II PI 5-phosphatases include the synaptojanins OCRL1, INPP5B, INPP5J, and SKIP. The type III enzymes are two SHIPs, namely SHIP1 and 2. Interestingly, there is only one type IV PI 5-phosphatase, named INPP5E. Because *E. histolytica* has a low conservation of PI 5-phosphatases, we will describe the search result at the end of this section. However, the enzyme Sac can act as a PI 5-phosphatase. Although it does not contain the 5-Ptase domain, it contains the CX_5_R motif-containing phosphatase domain (CKAGRSR). As described below, like PTEN, it differs from PI 5-phosphatases in its active center configuration and substrate specificity.

### 9.1. Type II PI 5-Phosphatase

There are five kinds of type II PI 5-phosphatases, such as synaptojanins, OCRL1, INPP5B, INPP5J, and SKIP. They differ in structure and function.

#### 9.1.1. General Description of Synaptojanins

There are two synaptojanins in mammals, and each of them has multiple splice variants (McPherson et al., 1996). All the synaptojanins have a conserved 5-Ptase catalytic domain and N-terminal Sac domain. The Sac domain also has inositol phosphatase activity. The domain configuration of the C-terminal region varies in each splice form (Figure 4). Most synaptojanin splice forms encode proteins that contain a proline-rich (P) region, and SYNJ1-170 additionally has an asparagine-proline-phenylalanine (NPF) repeat. NPF repeat is involved in the association of these proteins with the endocytic protein Eps15 (Haffner et al., 1997). SYNJ2A is ubiquitously expressed, and SYNJ1-170 shows a broad tissue distribution. Interestingly, SYNJ1-145, SYNEJ2B1, and SYNEJ2B2 are highly expressed in the brain, and SYNEJ2B1 and 2 are also abundant in the testis (Nemoto et al., 2001). The 5-Ptase domain of all SYNJ1 and 2 proteins enables them to dephosphorylate Ins(1,4,5)P_3_, Ins(1,3,4,5)P_4_, PtdIns(4,5)P_2_, and PtdIns(3,4,5)P_3_ at the D5 position (McPherson et al., 1996; Sakisaka et al., 1997). Furthermore, the Sac domain of Synaptojanins enable them to dephosphorylate PtdIns3P, PtdIns4P and PtdIns(3,5)P_2_ (Guo et al., 1999). Sac domain has been suggested to use the product generated by the PI 5-phosphatase domain to finally generate PtdIns (Guo et al., 1999; Nemoto et al., 2001). SYNJ1 has been found to be involved in synaptic vesicle exocytosis and recycling (McPherson et al., 1996). Additionally, synaptojanins have a critical role in the fate determination of clathrin-coated vesicles (CCVs). Knocking out SYNJ1 in the mouse caused accumulation of endocytosed CCVs and poor recycling of vesicles into the fusion-competent synaptic vesicle pool (Kim W. T. et al., 2002). Consequently, it caused neurological defects, and death after birth (Cremona et al., 1999). Synaptojanins are involved in endocytosis and synaptic vesicle recycling in concert with a variety of binding proteins such as endocytic proteins amphiphysin, endophilin syndapin/pacsin, and intersectin/Dap160 (McPherson et al., 1996; Bauerfeind et al., 1997; de Heuvel et al., 1997; Ringstad et al., 1997; Roos and Kelly, 1998; Qualmann et al., 1999). Also, the C-terminal NPF region of SYNJ1-170 enables the interaction with the EH (Eps15 homology) domain of Eps15, ear domain of the α-adaptin component of the adaptor protein (AP) 2 complex, and N-terminal domain of the clathrin heavy chain (Barbieri et al., 2001; Krauss et al., 2006).

#### 9.1.2. General Descriptions of OCRL1 and INPP5B

OCRL1 was originally identified as a protein responsible for the *X*-linked human disease OCRL. It shares a high amino acid sequence homology (45%) with INPP5B (Attree et al., 1992; Jefferson and Majerus, 1995; Speed et al., 1995; Matzaris et al., 1998). OCRL1 and INPP5B consist of a PH domain, 5-Ptase domain, ASH (ASPM, SPD2, hydin) domain, and RhoGAP domain. Only OCRL1 has two clathrin binding domains (CB), and only INPP5B has a C-terminal CAAX extension for prenylation of the cysteine residue (Jefferson and Majerus, 1995). These two proteins are the only RhoGAP domain-containing PI 5-phosphatases in the human and mouse (Jefferson and Majerus, 1995; Speed et al., 1995; Matzaris et al., 1998; Lowe, 2005). However, their RhoGAP domains appear to lack the catalytic activity because of amino acid substitutions at the catalytic region (Peck et al., 2002). Even though these domains lack a catalytic activity, RhoGAP domain of OCRL1 can interact with Rac1, Cdc42, ARF1, and ARF6 (Faucherre et al., 2003; Lichter-Konecki et al., 2006; Erdmann et al., 2007; Choudhury et al., 2009). OCRL1 prefer PtdIns(4,5)P_2_, Ins(1,4,5)P_3_, Ins(1,3,4,5)P_4_, and PtdIns(3,4,5)P_3_ as dephosphorylation substrates (Zhang et al., 1995; Schmid et al., 2004). OCRL1 can also dephosphorylate PtdIns(3,5)P_2_ (Schmid et al., 2004). On the other hand, INPP5B can remove the D5 phosphate of PtdIns(4,5)P_2_, PtdIns(3,4,5)P_3_, Ins(1,4,5)P_3_, and Ins(1,3,4,5)P_4_ with comparable efficiencies, but it is ineffective in the dephosphorylation of PtdIns(3,5)P_2_ (Jefferson and Majerus, 1995; Schmid et al., 2004). OCRL1 is involved in the regulation of vesicular trafficking between endosomes and the TGN (Choudhury et al., 2005). This is in line with OCRL1's localization on the TGN, early endosomes, membrane ruffles, and clathrin-coated trafficking intermediates (Olivos-Glander et al., 1995; Dressman et al., 2000; Ungewickell et al., 2004; Choudhury et al., 2005; Faucherre et al., 2005; Erdmann et al., 2007). OCRL1 deficiency causes abnormal cytoskeletal reorganization (Suchy and Nussbaum, 2002; Faucherre et al., 2005). This is not only due to perturbation of the functions exerted by the RhoGAP-like domain, but also due to perturbed modulation of PtdIns(4,5)P_2_ levels, suggesting the importance of OCRL1 in PtdIns(4,5)P_2_ homeostasis. INPP5B is associated with the Golgi apparatus, ER exit sites, and Rab (Williams et al., 2007). Its localization is affected by interactions with Rab proteins (Vyas et al., 2000; Shin et al., 2005; Williams et al., 2007). These data suggest that INPP5B is involved in the early secretory pathway.

#### 9.1.3. General Descriptions of INPP5J and SKIP

INPP5J and SKIP (skeletal muscle- and kidney-enriched inositol polyphosphate phosphatase) have been identified by virtue of two conserved consensus motifs characteristic of PI 5-phosphatases (Mochizuki and Takenawa, 1999; Gurung et al., 2003). INPP5J is also called proline-rich inositol polyphosphate 5-phosphatase (PIPP) because of the two proline-rich regions at the N- and C- terminal of the protein. The N-terminal proline-rich region of INPP5J contains a putative SH3-binding motif (PRSPSR) and six 14–3–3 binding motifs (Gurung et al., 2003), and a SKICH (SKIP carboxyl homology) domain which mediates localization to the plasma membrane (Ooms et al., 2006). The C-terminus of the 5-Ptase domain is overall conserved in both INPP5J and SKIP. INPP5J removes the D5 phosphate of PtdIns(4,5)P_2_, PtdIns(3,4,5)P_3_, Ins(1,4,5)P_3_, and Ins(1,3,4,5)P_4_, while SKIP prefers PtdIns(3,4,5)P_3_, but can also dephosphorylate PtdIns(4,5)P_2_, Ins(1,4,5)P_3_, and Ins(1,3,4,5)P_4_ (Ijuin and Takenawa, 2003). INPP5J and SKIP are localized on the plasma membrane, and also in the TGN and ER of resting cells, respectively. Following insulin stimulation, SKIP translocates to the plasma membrane via the SKICH domain (Gurung et al., 2003). Both enzymes are considered to regulate Akt activation by negatively regulating PtdIns(3,4,5)P_3_ (Ijuin and Takenawa, 2003; Ooms et al., 2006). Furthermore, SKIP participates in insulin response by regulating GULT4 translocation and glucose uptake in skeletal muscle.

### 9.2. Type III PI 5-Phosphatase

Type III PI 5-phosphatases, termed SHIP (SH2-containing inositol phosphatase), consists of SHIP1 and SHIP2. SHIP1 has several splice variants: a full-length isoform (SHIP1α) and shorter isoforms (SHIP1β, SHIP1γ, and s-SHIP1) (Lucas and Rohrschneider, 1999; Tu et al., 2001). SHIP1 was originally identified as a binding protein of several adaptor proteins such as Shc, Grab2, and DOK (Liu et al., 1994), and immunoreceptor tyrosine-based inhibitory (ITIM) or activation (ITAM) motifs of immune receptors (Osborne et al., 1996; Kimura et al., 1997). Both enzymes are composed of an N-terminal SH2 domain, a 5-Ptase catalytic domain followed by a C2 domain, and an NPXY motif. All SHIP isoforms except for SHIP1γ contain a C-terminal proline-rich domain. SHIP2 has a sterile alpha (SAM) motif at the C-terminus of the proline rich region. SHIP1 is exclusively expressed in hematopoietic and spermatogenic lineages, whereas SHIP2 expression is ubiquitous (Liu et al., 1998). SHIP1 isoforms hydrolyze the D5 phosphate of both PtdIns(3,4,5)P_3_ and Ins(1,3,4,5)P_4_, whereas SHIP2 activity appears to be more specific for PtdIns(3,4,5)P_3_ (Wisniewski et al., 1999). SHIP1 is involved in myeloid cell homeostasis, chemotaxis, bone metabolism, and mast cell activation (Helgason et al., 1998; Huber et al., 1998; Liu et al., 1999; Jiang et al., 2005; Nishio et al., 2007). SHIP2 is involved in the negative regulation of insulin-mediated cellular response (Wada et al., 2001; Kaisaki et al., 2004; Sleeman et al., 2005). These phenotypes are explicable given the high level of PtdIns(3,4,5)P_3_ in the cells, also pointing to the pivotal role of SHIPs in PtdIns(3,4,5)P_3_-mediated signaling.

### 9.3. Type IV PI 5-Phosphatase

There is only one type IV PI 5-phosphatase, which is also called INPP5E, 72-kDa polyphosphate 5-phosphatase, or Pharbin (Kisseleva et al., 2000; Kong et al., 2000). INPP5E consists of the C-terminal farnesylation CAAX motif and the central 5-phosphatase domain, flanked by an N-terminal proline-rich region. PtdIns(4,5)P_2_, PtdIns(3,4,5)P_3_, and PtdIns(3,5)P_2_ have been reported to be substrates of INPP5E (Kisseleva et al., 2000; Kong et al., 2000). Notably, INPP5E has the highest affinity to PtdIns(3,4,5)P_3_ and shows ten times higher affinity for PtdIns(3,4,5)P_3_ than SHIP (Kisseleva et al., 2000). It localizes to the cytosol, and Golgi in proliferating cells (Kong et al., 2000). In macrophages, INPP5E localizes to the phagocytic cup and regulates FcγR1-mediated, but not complement receptor 3-mediated, phagocytosis (Horan et al., 2007). In adipocytes, INPP5E has been shown to hydrolyze PtdIns(3,5)P_2_ to PtdIns3P and enhance GULT4 translocation; however, insulin signaling does not decrease upon INPP5E overexpression (Kong et al., 2000). Importantly, INPP5E also plays a critical role in ciliopathies (Bielas et al., 2009; Jacoby et al., 2009). The primary cilium is a microtubule-based organelle, which forms protrusions on the plasma membrane and functions as a sensor for the environmental factors such as light, chemicals, and mechanical stress (Goetz and Anderson, 2010). Additionally, primary cilia are central in hedgehog signaling, which is a major pathway involved in the structural organization of the body, organogenesis, and tumorigenesis (Elliott and Brugmann, 2019). PtdIns4P and PtdIns(4,5)P_2_ have been shown to be localized on the primary cilium membrane and ciliary base, respectively. This position-specific PI distribution pattern is maintained by INPP5E localized at the primary cilium (Chávez et al., 2015; Garcia-Gonzalo et al., 2015). Disrupting the PI compartmentalization pattern or increasing the PtdIns(4,5)P_2_ level in the primary cilium causes cilial accumulation of Tulp3, which contains a PtdIns(4,5)P_2_-binding domain. In turn, Tulp3's binding proteins, Gpr161 and IFT-A, are recruited to the primary cilium. Gpr161 is a negative regulator of the hedgehog signaling, and IFT-A is a flagellar transporting protein, respectively. Accumulation of this complex has been shown to perturb hedgehog signaling from the primary cilium. This observation emphasizes the significance of PI metabolism in cell physiology (Nakatsu, 2015).

### 9.4. PI 5-Phosphatases of *E. histolytica*

The possible *E. histolytica* PI 5-phosphatases all have a simple domain configuration: a 5-phosphatase domain alone or together with one more domain (Figure 4). PI 5-phosphatases have divergent and specialized roles in the human. For instance, synaptojanins are involved in neurotransmitter secretion, while SHIPs participate in hematopoietic cell signaling. Thus, the simplification of 5-phosphatases in *E. histolytica* may indicate the importance of IP5-phosphatase in multicellularity, and also the possibility of *E. histolytica* having only the ancestral type of PI 5-phosphatases.

Six candidate proteins showed E-values lower than 1 × 10^−30^ when searched with human OCRL1 as the query (AAB03839). One candidate, EHI_153490, has the 5-Ptase and RhoGAP domains, and the other protein, EHI_159880, contains the 5-Ptase and GAP domains. They both lack the C-terminus CAAX motif conserved in INPP5B. The apparent similarity in domain configurations suggests that these two proteins may have homologous roles to those of OCRL1. The rest of the hits (four) only have the 5-Ptase domain (Supplementary Figure S3) and showed only low levels of E-values to OCRL1 (Supplementary Table S5). Searching with type III and type IV PI 5-phosphatases detected the six candidates with higher E-values. Based on this result, we tentatively assigned the four proteins as type II PI 5-phosphatases homologous to OCRL1 (Figure 4; Supplementary Table S2). When searched with synaptojanin1 and synaptojanin2, nine candidates with E-values lower than 1 × 10^−25^ were detected. Six of them are identical to the above mentioned OCRL1 homologs and showed lower E-values to OCRL1. Other three candidates have a SAC domain, and thus they are considered as SAC homologs (see section 10). EHI_040380, as one of these SAC domain-containing proteins, was found to also harbor a DNase I domain by InterPro analysis (https://www.ebi.ac.uk/interpro/). The domain is classified in the endonuclease/exonuclease/phosphatase superfamily (IPR036691). As mentioned above, the 5-Ptase domain shares homology to the apurinic/apyrimidinic family of endonucleases (Whisstock et al., 2000), but EHI_040380 lacks the residues critical for the 5-Ptase activity (Supplementary Figure S3). Consequently, we classified this domain as “SAC with DNase I or 5-Ptase” domain (Figure 4). If this protein has a IP 5-phosphatase activity, it may act as synaptojanin in *E. histolytica*.

## 10. Sac Family Phosphatases

### 10.1. General Description of Sac

The first member of Sac (suppressor of actin), Sac1, was originally discovered in yeast by two independent genetic suppressor screens, which searched for the genes that rescued either actin cytoskeleton defects (Novick et al., 1989) or secretion defects caused by *sec14* mutation (Cleves et al., 1989). The catalytic domain of the Sac family phosphatases conserves the CX_5_R motif, which is commonly found in protein and PI phosphatases. According to the crystal structure, configuration of Sac1 catalytic center has a unique feature compared with PTEN and MTMs (Lee et al., 1999; Begley et al., 2003, 2006; Manford et al., 2010). The catalytic cysteine is oriented away from the conserved arginine in Sac1, while the corresponding residue in PTEN and MTMs faces toward the arginine and generates a narrow active center. This observation suggests that Sac1's catalytic center probably undergoes a conformational change during the catalysis. This premise appears to agree with the fact that Sac1 is an allosteric enzyme, and its activity is stimulated by anionic phospholipids (Zhong et al., 2012). The Sac domain is also found in synaptojanins (see section 9.1). It is responsible for the removal of phosphate from D3, D4, and/or D5 positions of various PIs, and thus, Sac is considered to be a PI phosphatase with broad specificity. Sac1 dephosphorylates PtdIns3P, PtdIns4P, and PtdIns(3,5)P_2_, but not PtdIns(4,5)P_2_ (Nemoto et al., 2000), and Sac2 acts on D5 position of PtdIns(4,5)P_2_ and PtdIns(3,4,5)P_3_ (Minagawa et al., 2001), whereas Sac3 hydrolyzes only PtdIns(3,5)P_2_ (Botelho et al., 2008). Sac1 is mostly localized on the ER and shuttles between the Golgi and ER. The C-terminus of Sac1 mediates an association with the COPI complex via a conserved KXKXX motif and this association induces the retrieval of Sac1 to the ER (Blagoveshchenskaya et al., 2008). Sac1 preferentially utilizes PtdIns4P as its substrate, and mutations that downregulate Sac1 cause the cellular PtdIns4P levels to increase in yeast and mammals (Guo et al., 1999; Nemoto et al., 2000). Sac1 has been also shown to be involved in the maintenance of the plasma membrane PtdIns4P levels at the ER-plasma membrane junctions (Stefan et al., 2011). Sac2 is a mammalian-specific negative regulator of the Akt pathway (Trivedi et al., 2007), and involved in the endocytic pathway as a PI 4-phosphatase (Nakatsu et al., 2015). Sac3 is important for the regulation of the endocytic pathway given that it regulates PtdIns(3,5)P_2_ levels. Sac3 deficiency causes PtdIns(3,5)P_2_ levels to increase and impairs late-endosome to lysosome transition. It is also involved in the regulation of PIPKIII enzymatic activity (see section 6.3.1).

### 10.2. Sac of *E. histolytica*

In the *E. histolytica* genome database, three proteins (EHI_141860, EHI_040380, EHI_048570) showed E-values lower than 1 × 10^−10^ with human Sac1 in a blastp search. These proteins were also detected when Sac2 and Sac3 were used as queries, and the E-values were lower than those obtained with Sac1. Therefore, these three proteins seem to be homologous to the human Sac1. EHI_141860 has the highest homology to the human Sac1, conserves the two transmembrane domains (Figure 4), and thus, it is considered to be the *E. histolytica* Sac1 ortholog. EHI_141860 also has the C-terminal COPI complex binding motif, KXKXX. As mentioned above, EHI_040380 has a DNaseI domain. Since 5-Ptase domain shares homology to the apurinic/apyrimidinic family of endonucleases (Whisstock et al., 2000), there is a possibility that this protein functions like synaptojanin. However, as the domain in EHI_040380 lacks the residues critical for the active center of PI 5-phosphatase (Supplementary Figure S3), we classified it as “SAC with DNase I or PI 5-phosphatase” domain (Figure 4). Nevertheless, further experimental evidence on its PI 5-phosphatase potential is necessary. Since *E. histolytica* appears to lack PI 4-phosphatases (see section 8), Sac orthologs may act as PI 4-phosphatase in this organism.

## 11. Possible Pathway-dependent Coordinated Regulation of Key PI Metabolizing Enzymes

### 11.1. Biosynthetic, Secretory, and Exocytotic Pathways

While a majority of interconversion steps between specific PIs are catalyzed by >1 enzymes, some steps are catalyzed by a single enzyme encoded by a single gene, which may suggest the significance of the enzyme. Furthermore, mRNA expression levels, inferred by transcriptomic analyses, often suggest biological importance of the enzyme(s) for the reaction under given conditions. In the biosynthetic and secretory pathways, the PtdIns4P level is maintained in an organelle specific fashion: low in the ER and high in the Golgi, by the coordinated action of PI 4-kinases, PI4KIIα and PI4KIIIβ, and Sac1 (Blumental-Perry et al., 2006; Graham and Burd, 2011; Bajaj Pahuja et al., 2015). *E. histolytica* possesses only one PI 4-kinase, EHI_148700, which is most likely involved in this pathway. Role of Sac1 to maintain the low PtdIns4P level in the ER was shown (Bajaj Pahuja et al., 2015). It is conceivable to assume a single two transmembrane domain-containing PI-phosphatase Sac, EHI_141860, likely plays important role to maintain the low PtdIns4P level in the Golgi to regulate biosynthetic and secretory pathways. The transcriptome data also suggest the robust expression (Supplementary Figure S1) and thus the significance of EHI_141860.

Since clathrin-mediated trafficking machinery for the exocytic pathway is well-conserved in this organism (Clark et al., 2007), PtdIns4P is likely used in the Golgi apparatus as a key signaling molecule to recruit effector molecules, such as clathrin binding proteins AP, Arf, and Rab11for cargo selection and packaging. Following the generation of transport vesicles in the Golgi, PtdIns4P on the PtdIns4P-rich transport vesicles is replaced with sterol by oxysterol binding protein (OSBP), to form sterol-rich vesicles (Schink et al., 2016). This exchange is necessary to recruit exocyst complex onto the transfer vesicles and also function as sterol transfer mechanism from the ER to the plasma membrane (Schink et al., 2016). It remains elusive if this mechanism also works in *E. histolytica*, although its genome encodes 2-4 possible OSBP (Das and Nozaki, 2018).

At the plasma membrane, PtdIns(4,5)P_2_ generated from PtdIns4P by single type I PIP kinase, EHI_153770, likely determines the site of exocytosis, where the exocyst complex mediates a release of the content of the sterol-rich secretory vesicles. Sec3 and Exo70 of the exocyst complex are known PtdIns(4,5)P_2_ effectors on secretory vesicles. On the plasma membrane, Syntaxin-1, CAPS, Munc13-1/2, and Synaptotagmin-1 involved in the fusion of transport vesicles and the plasma membrane, leading to secretion (Martin, 2015). *E. histolytica* conserves a homolog of Syntaxin (EHI_052830, E-value 1 × 10^−18^), Sec3 (EHI_148590, E-value 9 × 10^−6^), and Exo70 (EHI_142040, E-value 2 × 10^−8^). The functionality of the apparently conserved basic amino acids implicated for PtdIns(4,5)P_2_ binding should be verified for the amebic homolog (Martin, 2015; K. Nakada-Tsukui data not shown).

### 11.2. Endocytic Pathways

In the clathrin-dependent endocytic pathway, AP complex connects membrane cargo receptors and clathrin via PtdIns(4,5)P_2_ by recognizing the cytoplasmic region of the cargo receptors and PtdIns(4,5)P_2_. In this process, single type I PIP kinase, EHI_153770, is necessary to synthesize PtdIns(4,5)P_2_ from PtdIns4P. During the scission of the vesicles from the plasma membrane, generation of PtdIns(3,4)P_2_ from PtdIns4P by class II PI 3-kinase is necessary to recruit SNX9 (sorting nexin that recognizes membrane curvature and PIs) in mammals. However, *E. histolytica* does not possess either PX-domain containing class II PI 3-kinases or BAR domain containing SNXs, e.g., SNX9. However, it is conceivable that one or some of six class I PI 3-kinases also have PtdIns4P 3-kinase activity and some of putative SNXs lacking BAR domain have ability to recognize PIs (N. Watanabe et al., data not shown). Therefore, it is expected that as enclosed endosomes mature after closure, PtdIns(4,5)P_2_ is subsequently dephosphorylated into PtdIns and then further phosphorylated to PtdIns3P by the action of type III PI 3-kinase, Vps34, which is present in *E. histolytica* as a single protein. In mammals, a series of dephosphorylation reactions involving PtdIns(4,5)P_2_ are regulated by OCRL1 and Sac2 (Nakatsu et al., 2015) and Synaptojanins (Cremona et al., 1999). Since Synaptojanins, which contain PI 5-phosphatase and PI 4-phosphatase domains, are not conserved in *E. histolytica*, the most highly transcribed PI 5-phosphatase, EHI_160860, out of 6 type II PI 5-phosphatases, and one of two transmembrane lacking Sac proteins, EHI_040380 and EHI_048570, are likely involved in this process (Figure 4; Supplementary Figure S1).

### 11.3. Phago- and Trogocytic Pathways

During phagocytosis, one single type I PIP-kinase, EHI_153770, and/or one or more of six class I PI 3-kinases are likely involved in local enrichment of PtdIns(4,5)P_2_ and PtdIns(3,4,5)P_3_ from PtdIns4P in response to the signal from a not-yet-identified ligand receptor (most likely galatose/N-acetylgalactosamine specific lectin) for phagocytosis at the plasma membrane. Based on the mRNA expression levels, five out of 6 class I PI 3-kinase genes appear to be abundantly expressed at similar levels and thus, it is not clear which is predominantly involved in this process (Supplementary Figure S1). In mammalian THP-1 cells, isoform-specific roles of class I PI 3-kinases were reported: p110α is involved in FcγR-mediated phagocytosis and oxidative burst mediated by PMA or opsonized zymosan, but not in CR3-mediated phagocytosis (Lee et al., 2007). On the other hand, p110β is involved in Rab5 recruitment and activation during phagosome maturation, while p110δ is involved in adhesion to VCAM-1 (Kurosu and Katada, 2001; Ferreira et al., 2006; Thi et al., 2012; Whitecross and Anderson, 2017).

In *E. histolytica*, two different modes of ingestion for target uptake, phagocytosis and trogocytosis, have been observed, and they have been shown to be regulated by different AGC kinases in an isotype-specific manner. It is conceivable that different receptors and class I PI 3-kinases are differentially involved in these processes. Elucidation of the isoform-specific involvement of class I PI 3-kinases in trogocytosis and phagocytosis shall be important to understand the pathogenesis of *E. histolytica*.

On the enclosed phagosomes, PtdIns(4,5)P_2_ and PtdIns(3,4,5)P_3_ are, as also seen in endocytosis, dephosphorylated to PtdIns by PI-phosphatases and then phosphorylated again to form PtdIns3P by class III PI 3-kinase, such as OCRL1, NPP5B, SHIP, INPP5E, TMEM55a, myotubularin, Sac2, and Vps34, in mammals (Cox et al., 2001; Ai et al., 2006; Horan et al., 2007; Neukomm et al., 2011; Bohdanowicz et al., 2012; Levin et al., 2017; Morioka et al., 2018). Similarly, conversion of PIs on the *E. histolyica* phagosomes is expected to proceed in a similar but possibly modified fashion. Among a number of type II PI 5-phosphatases, Sacs, myotubularins, and type III PI 3-kinase, we assume the following members are likely involved in this process. Among eight myotubularins that appear to be catalytically active, three isotypes, EHI_016430, EHI_104710, and, EHI_024380, show relatively high expression and are likely involved in this maturation process. Once phagosomes are decorated with PtdIns3P, PtdIns3P is further phosphorylated to PtdIns(3,5)P_2_ as phagosomes are further maturated. While in mammals, PIKfyve is involved in this process, type III PIP-kinase lacking FYVE domain, EHI_049480, may be responsible for this reaction in *E. histolytica*. As described above (section 6.3.1), type III PIP-kinases form complex with Sac phosphatase and scaffold proteins (Sbrissa et al., 2007; Botelho et al., 2008; Jin et al., 2008). Since two Sacs lacking the transmembrane domain, EHI_040380 and EHI_048570, are present in *E. histolytica*, it is possible that one of them forms complex with EHI_049480, similar to mammalian Sac3, while the other independently works in endocytic and phagocytic pathways similar to mammalian Sac2 (Nakatsu et al., 2015; Levin et al., 2017).

### 11.4. Motility

In the regulation of cell motility, local accumulation of PtdIns(4,5)P_2_ and PtdIns(3,4,5)P_3_ at the leading edge is the key initial event. Similar to phagocytosis, type I PIP-kinase, EHI_153770, and some of six type I PI 3-kinases are likely involved in this process. Also, dephosphorylation of PtdIns(3,4,5)P_3_ by PI 3-phosphatases, PTEN, and PI 5-phosphatase, SHIP, at the side and the rear of the cell is known to be indispensable to regulate local accumulation of the lipid signal in mammals. In *E. histolytica*, some of six PTEN homologs and six PI 5-phosphatases are likely involved in this process. Among three of six amebic PTENs that contain C2 domain and the putative cytosol localization signal (see section 7.1.2), two showed significantly higher expression levels than four other PTEN isotypes (Supplementary Figure S1). Altogether, these data suggest that these two PTENs, EHI_197010 and EHI_098450, may be involved in the formation and maintenance of cellular polarity in *E. histolytica*.

### 11.5. Nuclear Functions

It is conceivable that PI kinases and PIP phosphatases that contain the nuclear localization signal have specific roles in the nucleus such as chromatin regulation and transcription. Such PI kinases and PIP phosphatases include type I PIP-kinase, EHI_153770; PTEN, EHI_041900; MTM, EHI_070120; and PI 5-phosphatase, EHI_046590. Also, PTENs that lack the cytosol localization signal, EHI_041900, EHI_010360, and EHI_054460, may also be involved in nuclear functions.

## 12. Conclusion and Future Perspective

One of the hallmarks of *E. histolytica* as an invasive eukaryotic pathogen is its extremely active cell motility accompanied with elaborate cytoskeletal rearrangement and membrane traffic. To enable such activities, spaciotemporal regulation of PI-mediated signaling that controls transient association with effector molecules is indispensable and accomplished via concerted regulation of PI metabolism. The *E. histolytica* genome encodes the majority of PI kinases and PI phosphatases conserved in model organisms. Strikingly, significant diversity of PI 3-kinases and PI 3-phosphatases was observed in *E. histolytica*, as represented by a higher level of complexity of class I PI 3-kinases, PTEN, MTM/MTMR, and IMLRKs in this unicellular eukaryote relative to human, which has a 100 times larger genome. The dependence of *E. histolytica* on the complexity of the D3 phosphate metabolism emphasizes the significance of PtdIns(3,4,5)P_3_-centric pathways for pathogenesis and physiology of *E. histolytica*.

On the other hand, the regulatory subunit of PI kinases, except for class III PI 3-kinase, was not identified, suggesting that their regulatory mechanisms had been gained only in higher eukaryotes or had differently evolved in *E. histolytica* in a lineage-specific fashion. The latter was also observed as an example with PI 4-kinase regulator EFR3 which is not conserved between the yeast and human. PtdIns4P metabolism also appears to have uniquely evolved in *E. histolytica*. No PtdIns4P-specific phosphatases that show similarity to the canonical enzymes are conserved in *E. histolytica*. It harbors only one PIP kinase, type I PIP kinase, which generate PtdIns(4,5)P_2_ from PtdIns4P. PtdIns4P is known as one of the major PIs and is important as it is the precursor of the most abundant PI, PtdIns(4,5)P_2_. Since PtdIns(4,5)P_2_ is indispensable for the regulation of actin cytoskeleton-dependent processes, which is vital for the pathogenesis of the amoeba, type I PIP kinase appears to be a rational drug target. Uniquely expanded gene families, such as class I PI 3-kinases and PTENs, may also be potential drug target. However, multiple enzymes may have a redundant role as shown for mammalian PIPKI (see section 6.1.1).

Besides expansion, certain families of PI kinases and PI phosphatases in *E. histolytica* are structurally unique in the sense that they have simpler domain configurations, especially type III PIP kinases and PI phosphatases, relative to their human counterparts with a exceptions such as inactive myotubularin/LRR/ROCO/kinase (IMLRK). Lineage-specific expansions of PIP phosphatases are found in particular for OCRL1 type II PI 5-phosphatase and IMLRK, some of which should be listed in the roster of rational drug targets once their functions are determined.

Extremely higher expression levels of two PTEN and one PI 5-phosphatase genes relative to other genes involved in PI metabolism may reflect the importance of phosphatases rather than kinases in stopping the PI signals. PTEN has also been reported to function as a protein phosphatase, and thus, it is also possible that the high expression of PTEN is because it has roles other than PI signaling (Shinde and Maddika, 2016; Wozniak et al., 2017).

Furthermore, it has recently been demonstrated that besides the phosphorylation status, type pf the acyl-chains in the lipids are important in the regulation of lipid functions (Choy et al., 2017). In mammals, the predominant type found in PIs is 1-stearoyl-2-arachidonoyl (18:0/20:4) (Traynor-Kaplan et al., 2017). This acyl chain type is generated by lysocardiolipin acyltransferase (LYCAT) (Imae et al., 2012), whose deficiency persturbes PI-mediated-membrane traffic (Bone et al., 2017). Mechanisms underlying such acyl-dependent regulation of PI signaling and downstream cascades are just about to unveil in the lipid research field and should be explored in amebiasis research. Furthermore, a new family of lipid transport proteins that mediate the regulation of PI metabolisms in both the cytoplasm and the nucleus have been described (Das and Nozaki, 2018). It may also be of interest that several PI kinases and PI phosphatases as well as several PI species are localized in the nucleus. However, PI metabolism and physiological roles of PI in the nucleus is poorly understood.

## Author Contributions

All authors listed have made a substantial, direct and intellectual contribution to the work, and approved it for publication.

### Conflict of Interest Statement

The authors declare that the research was conducted in the absence of any commercial or financial relationships that could be construed as a potential conflict of interest.

## Supplementary Material

Supplementary Figure S1 (Image 1) Expression levels of PI kinases and PI phosphatases in *E. histolytica* trophozoites. Relative expression levels of indicated PI kinase and PI phosphatase genes of the HM-1:IMSS cl6 reference strain during *in vitro* cultivation. Signal intensity was normalized against the transcript of RNA polymerase II gene. Two bars represent the data from two independent experiments (Husain et al., 2011; Penuliar et al., 2015).

Supplementary Figure S2 (Image 2) Cytosolic localization signal in human and *E. histolytica* PTEN. The cytosol localization signal of human PTEN was aligned with the corresponding region of *E. histolytica* PTEN orthologs. Green underlines depict the key amino acids for the signal and the amino acids conserved in PTENs from human and *E. histolytica* are indicated with green boxes.

Supplementary Figure S3 (Image 3) Conservation of consensus amino acid sequences in PI 5-phosphatases. Amino acids of two consensus regions of PI 5-phosphatases, human OCRL1 and *E. histolytica* orthologs. Gray boxes indicate conserved amino acids.

Supplementary Figure S4 (1–4) (Images 4–7) Multiple alignment of class I PI 3-kinases. LKU domain and catalytic core are indicated by green and yellow bar, respectively.

Supplementary Figure S5 (1–3) (Images 8–10) Multiple alignment of PTEN. Catalytic core is indicated by yellow bar.

Supplementary Table S1 (Tab 1 in Table 1) Potential PI kinase orthologs in *E. histolytica. H. sapiens* enzymes used as queries, and E-values are shown.

Supplementary Table S2 (Tab 2 in Table 1) Potential PI phosphatase orthologs in *E. histolytica. H. sapiens* enzymes used as queries, and E-values are also shown.

Supplementary Table S3 (Tab 3 in Table 1) Similarity between *H. sapiens* and *E. histolytica* class I PI 3-kinases. *H. sapiens* isotypes used as queries, and E-values are also shown.

Supplementary Table S4 (Tab 4 in Table 1) Similarity between *H. sapiens* and *E. histolytica* MTM and MTMRs. *H. sapiens* isotypes used as queries, and E-values are also shown.

Supplementary Table S5 (Tab 5 in Table 1) Similarity between *H. sapiens* and *E. histolytica* PI 5-phosphatases.

Supplementary Table S6 (Tab 6 in Table 1) Abbreviation list.

## Abbreviations

see Supplementary Table S6.
