# Supplementary figures and images for: Phosphatidylinositol Kinases and Phosphatases in Entamoeba histolytica
Source: Front Cell Infect Microbiol. 2019 Jun 6;9:150. doi: 10.3389/fcimb.2019.00150 (PMC6563779; doi:10.3389/fcimb.2019.00150)

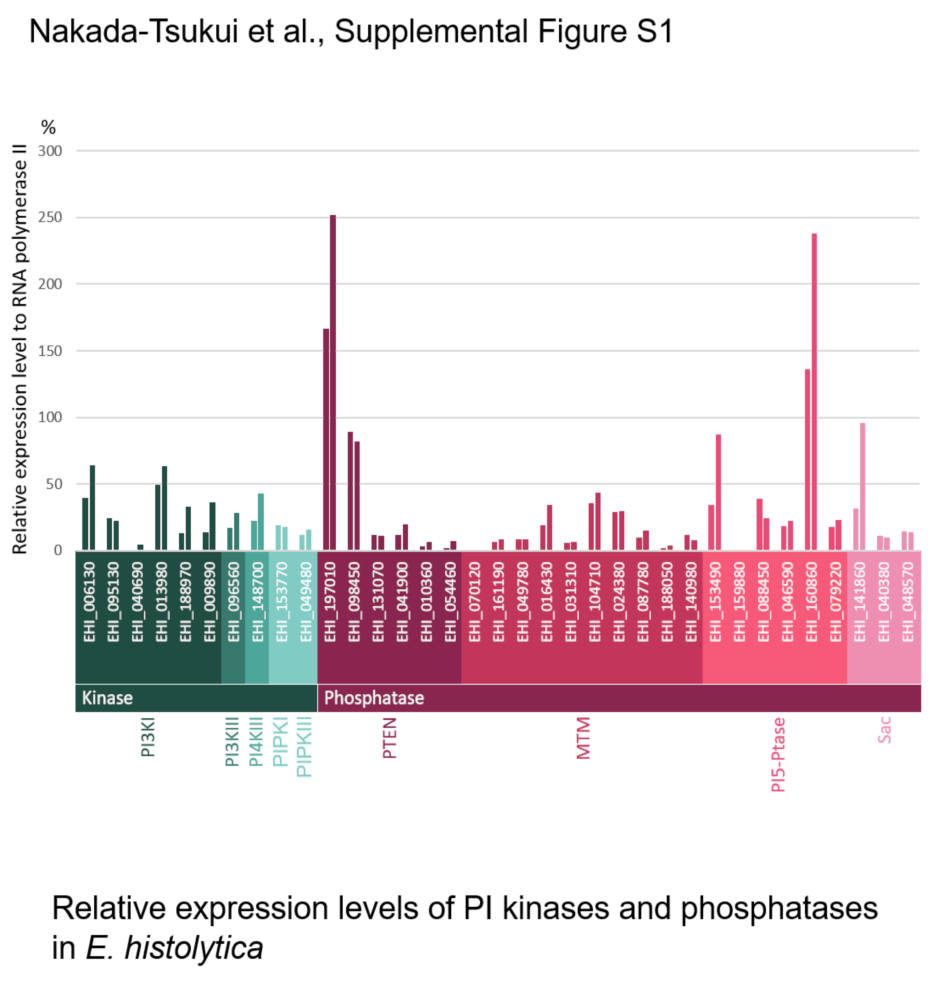

Supplement: Supplementary Figure S1 (Image 1) — Expression levels of PI kinases and PI phosphatases in E. histolytica trophozoites. Relative expression levels of indicated PI kinase and PI phosphatase genes of the HM-1:IMSS cl6 reference strain during in vitro cultivation. Signal intensity was normalized against the transcript of RNA polymerase II gene. Two bars represent the data from two independent experiments (Husain et al., 2011; Penuliar et al., 2015). [file Image_1.TIF]

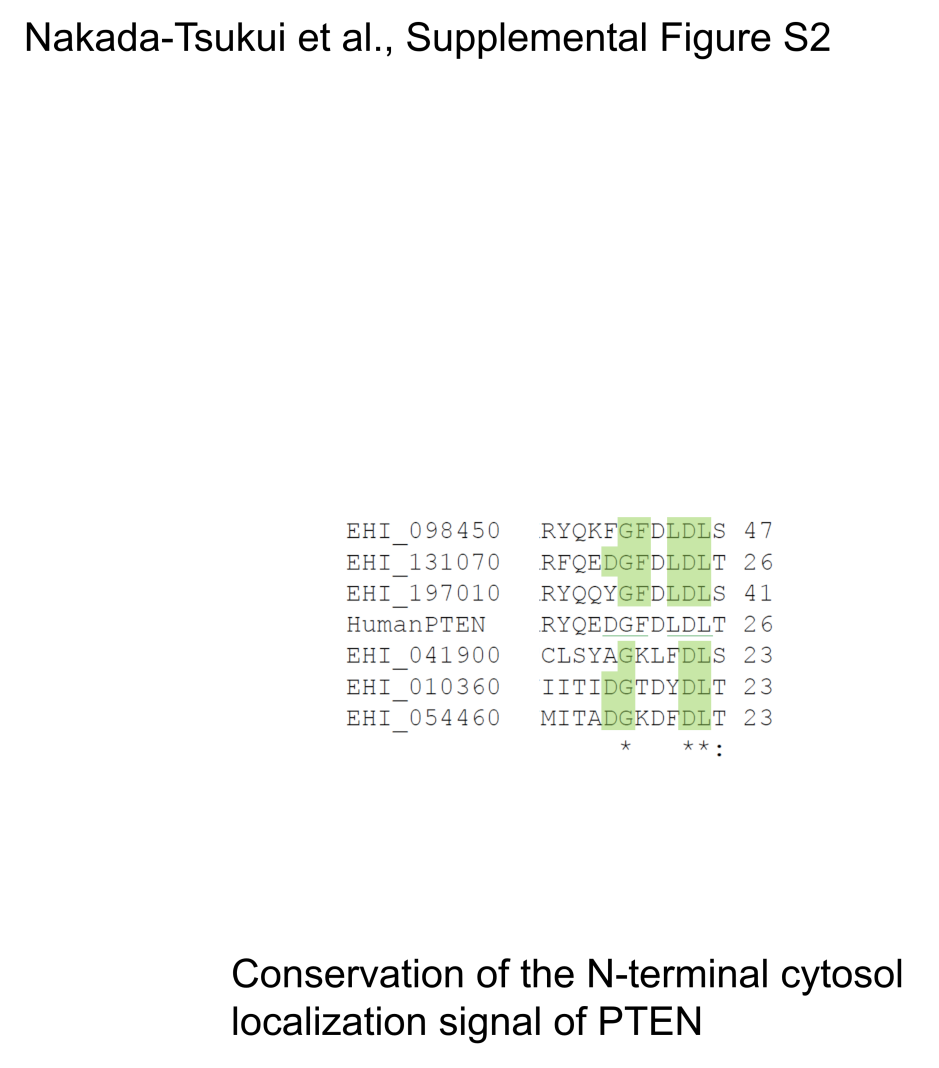

Supplement: Supplementary Figure S2 (Image 2) — Cytosolic localization signal in human and E. histolytica PTEN. The cytosol localization signal of human PTEN was aligned with the corresponding region of E. histolytica PTEN orthologs. Green underlines depict the key amino acids for the signal and the amino acids conserved in PTENs from human and E. histolytica are indicated with green boxes. [file Image_2.TIF]

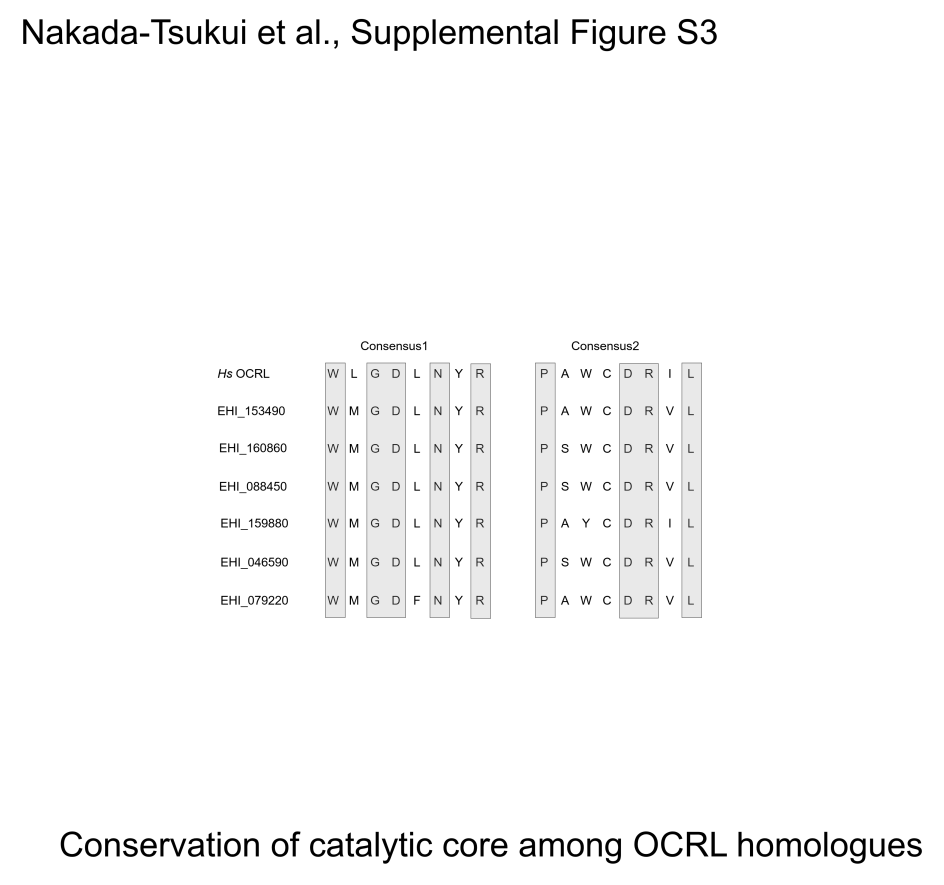

Supplement: Supplementary Figure S3 (Image 3) — Conservation of consensus amino acid sequences in PI 5-phosphatases. Amino acids of two consensus regions of PI 5-phosphatases, human OCRL1 and E. histolytica orthologs. Gray boxes indicate conserved amino acids. [file Image_3.TIF]

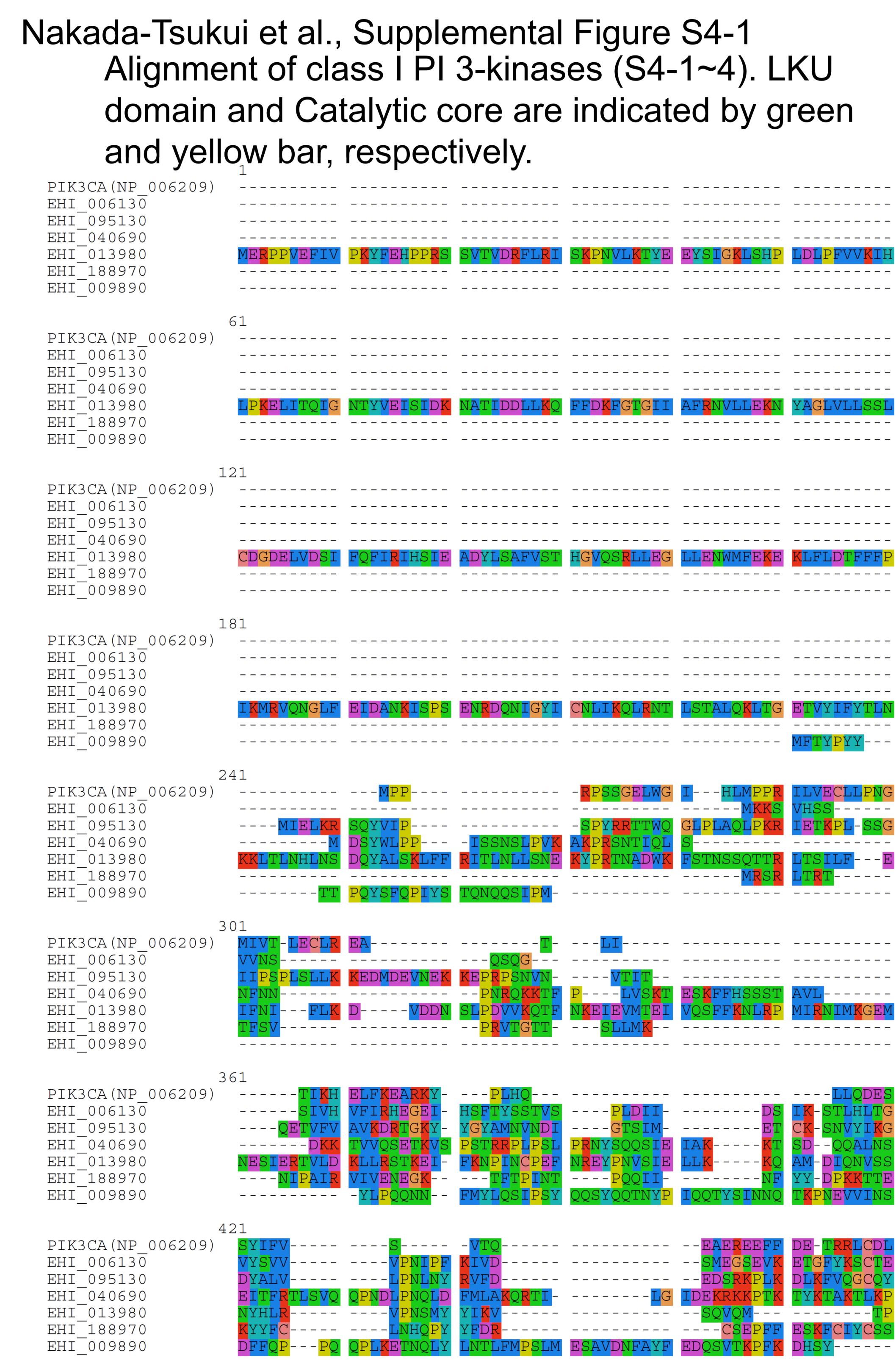

Supplement: Supplementary file 4 [file Image_4.TIF]

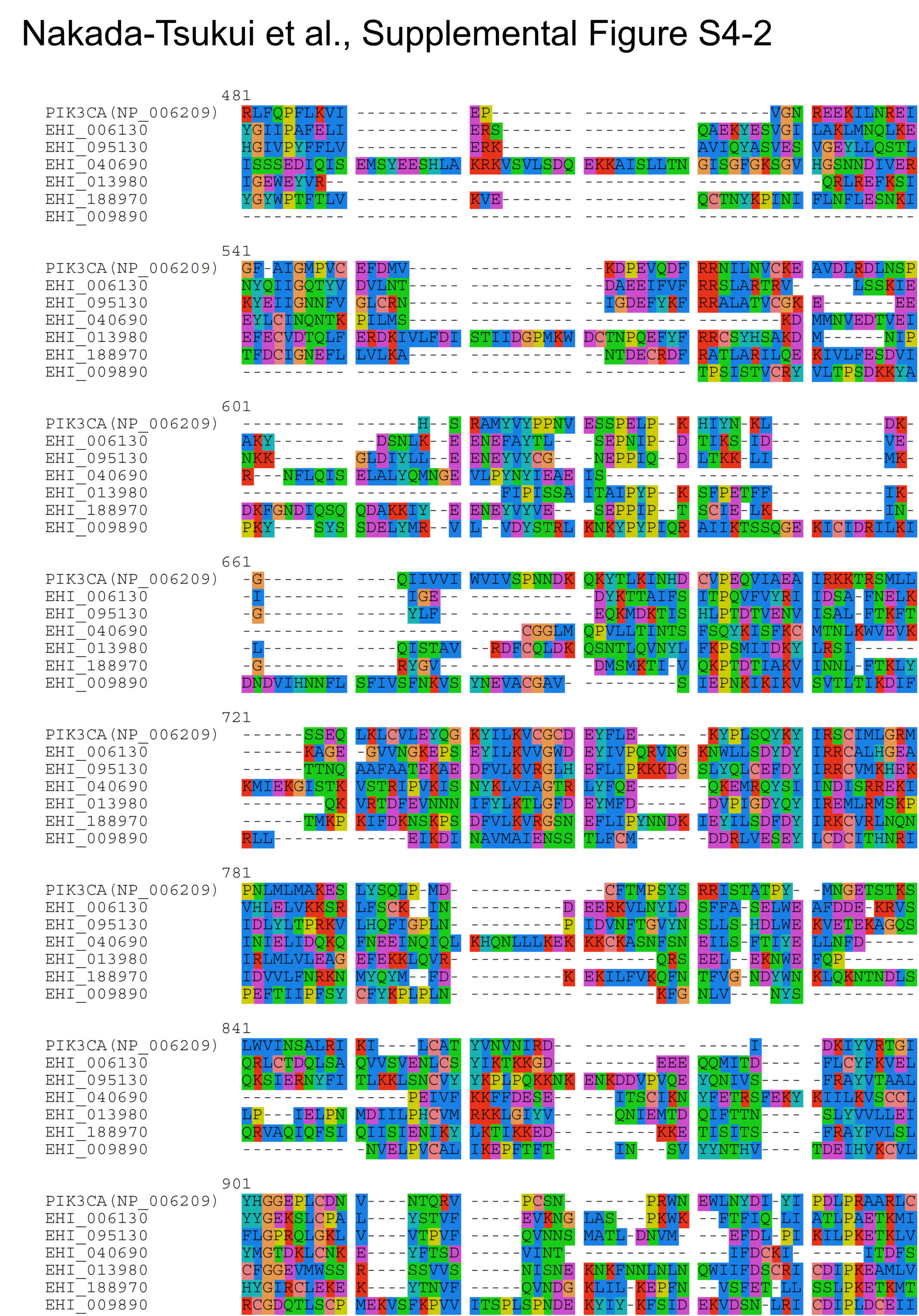

Supplement: Supplementary file 5 [file Image_5.TIF]

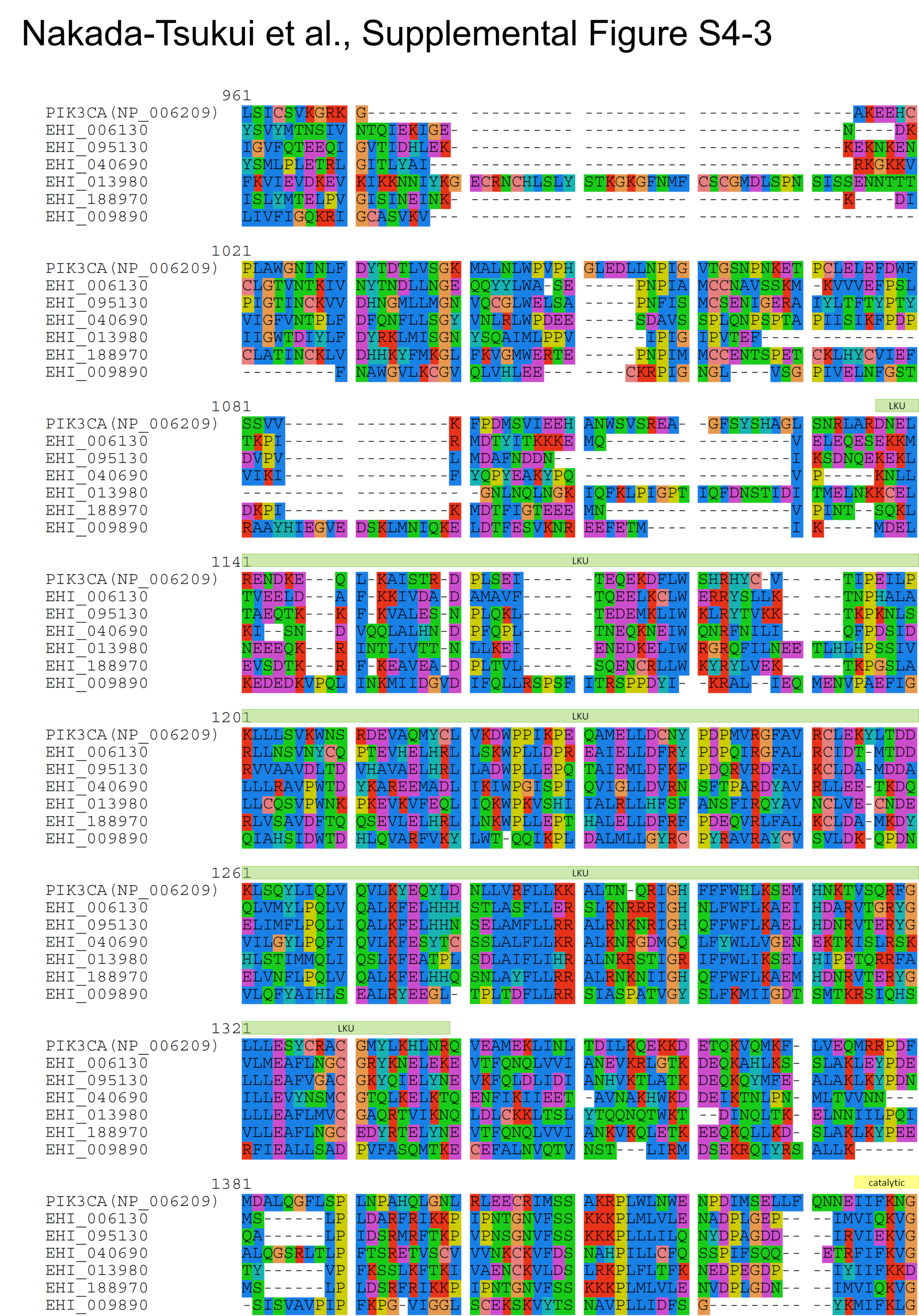

Supplement: Supplementary file 6 [file Image_6.TIF]

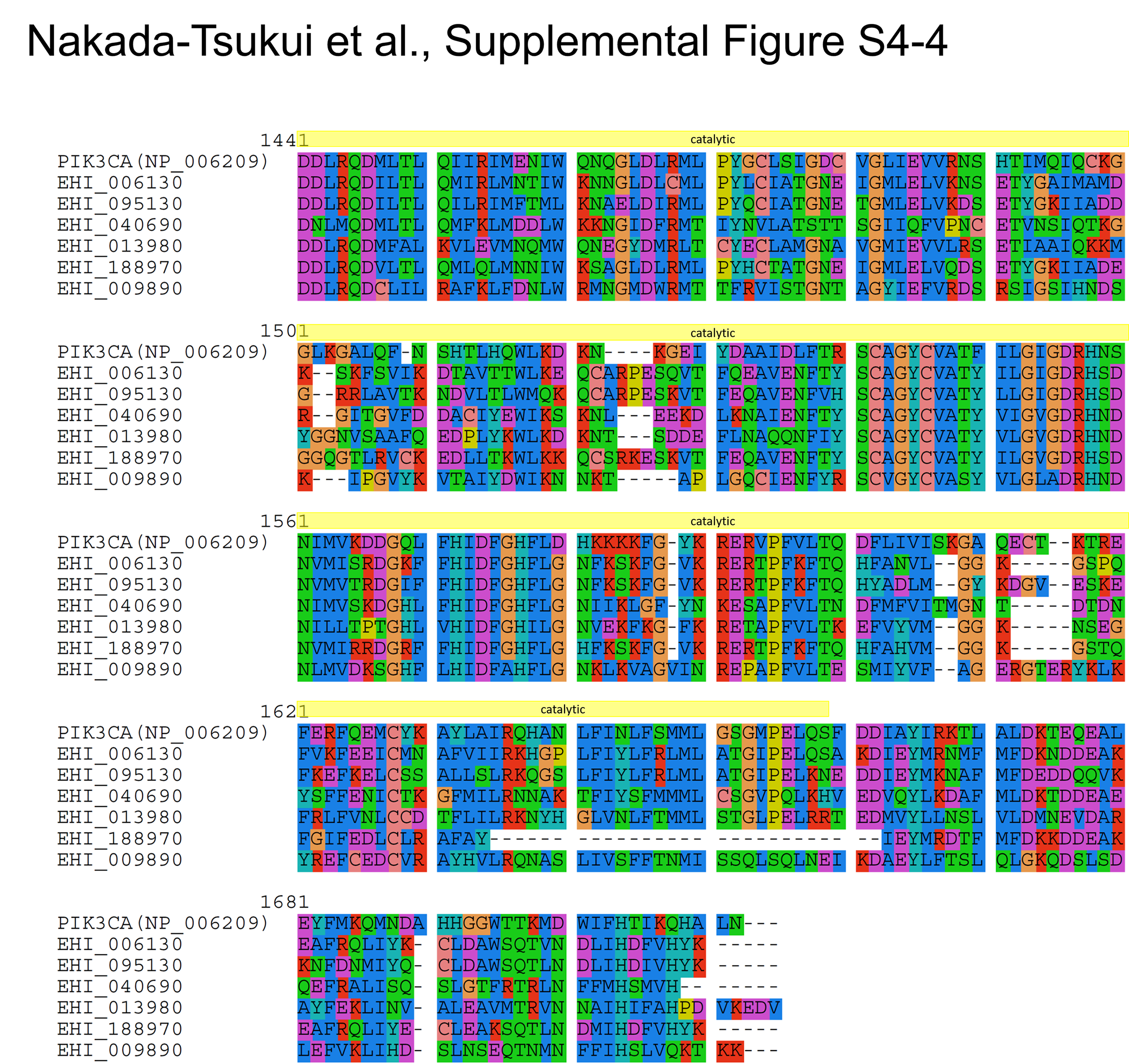

Supplement: Supplementary Figure S4 (1–4) (Images 4–7) — Multiple alignment of class I PI 3-kinases. LKU domain and catalytic core are indicated by green and yellow bar, respectively. [file Image_7.TIF]

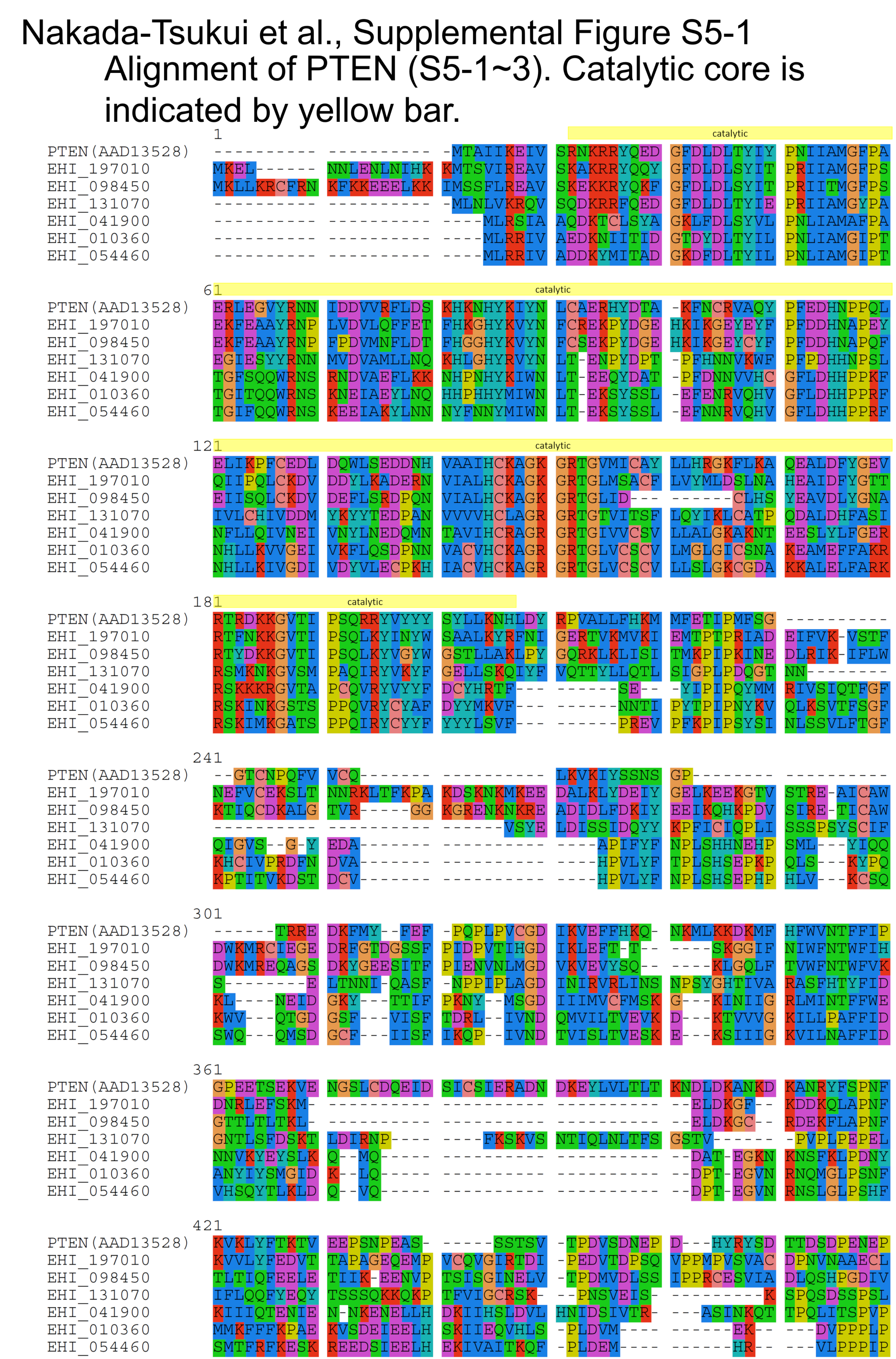

Supplement: Supplementary file 8 [file Image_8.TIF]

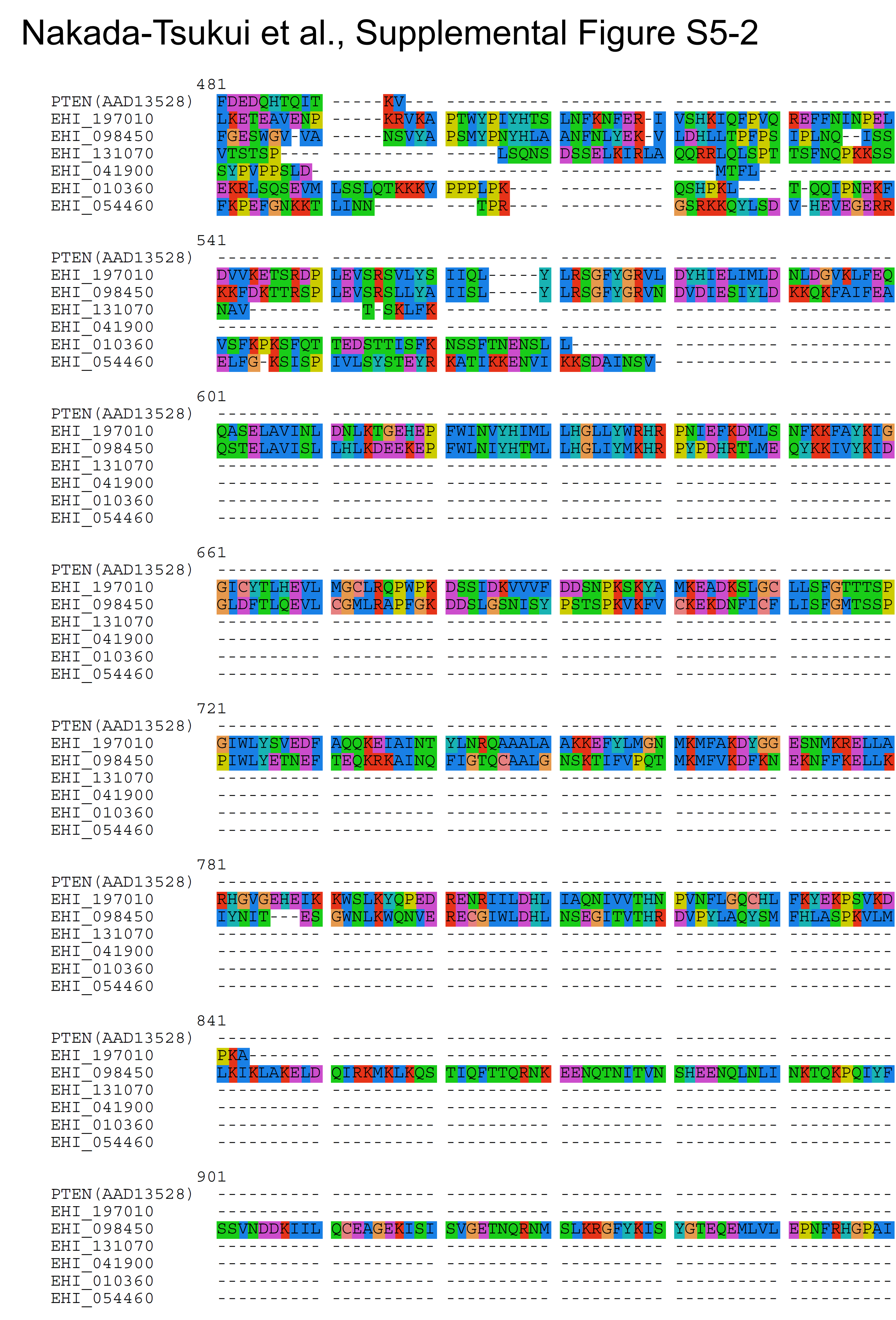

Supplement: Supplementary file 9 [file Image_9.TIF]

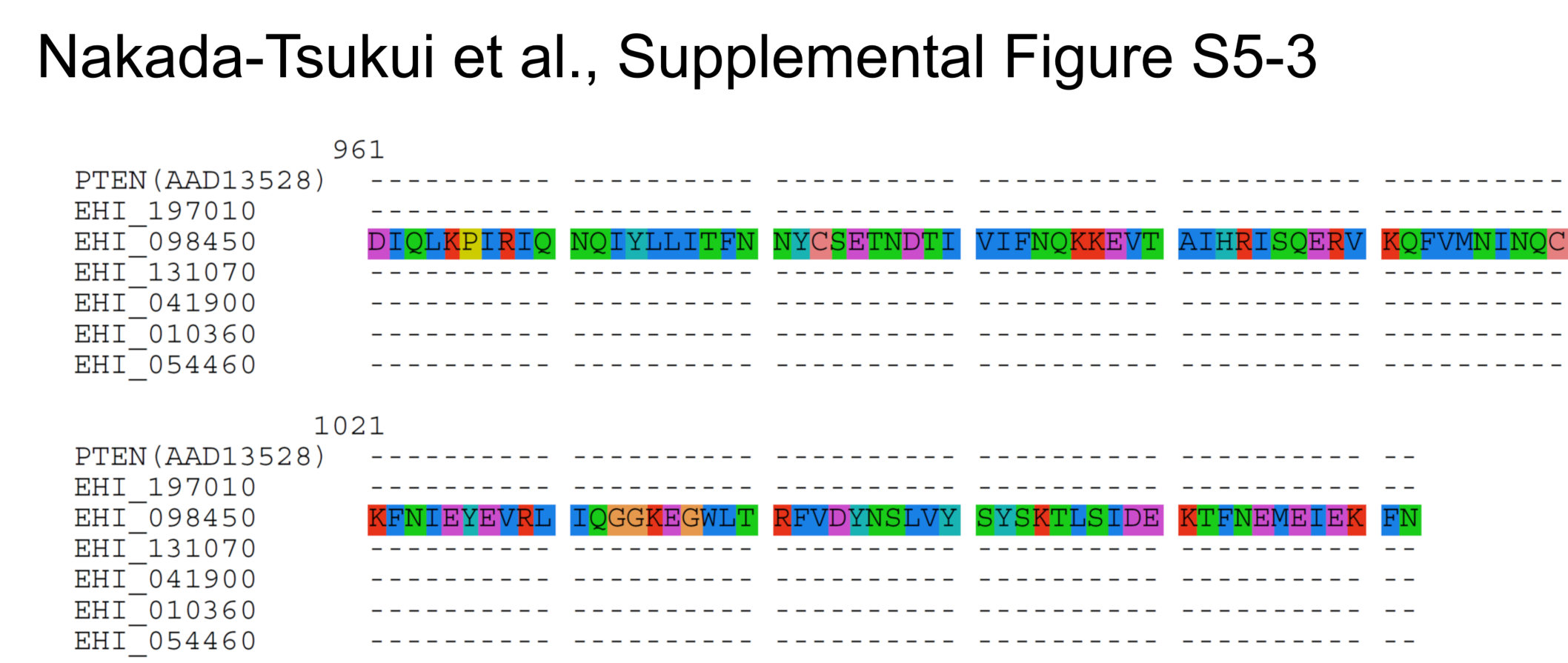

Supplement: Supplementary Figure S5 (1–3) (Images 8–10) — Multiple alignment of PTEN. Catalytic core is indicated by yellow bar. [file Image_10.TIF]
